# NTAL is associated with treatment outcome, cell proliferation and differentiation in acute promyelocytic leukemia

**DOI:** 10.1038/s41598-020-66223-2

**Published:** 2020-06-25

**Authors:** Carolina Hassibe Thomé, Germano Aguiar Ferreira, Diego Antonio Pereira-Martins, Guilherme Augusto dos Santos, César Alexander Ortiz, Lucas Eduardo Botelho de Souza, Lays Martins Sobral, Cleide Lúcia Araújo Silva, Priscila Santos Scheucher, Cristiane Damas Gil, Andréia Machado Leopoldino, Douglas R. A. Silveira, Juan L. Coelho-Silva, Fabíola Traina, Luisa C. Koury, Raul A. M. Melo, Rosane Bittencourt, Katia Pagnano, Ricardo Pasquini, Elenaide C. Nunes, Evandro M. Fagundes, Ana Beatriz F. Gloria, Fábio Rodrigues Kerbauy, Maria de Lourdes Chauffaille, Armand Keating, Martin S. Tallman, Raul C. Ribeiro, Richard Dillon, Arnold Ganser, Bob Löwenberg, Peter Valk, Francesco Lo-Coco, Miguel A. Sanz, Nancy Berliner, Vitor Marcel Faça, Eduardo M. Rego

**Affiliations:** 1 Department of Biochemistry and Immunology, Medical School of Ribeirão Preto, University of São Paulo, Ribeirão Preto, Brazil; 2 Regional Blood Center of Ribeirão Preto, Medical School of Ribeirão Preto, Center for Cell Based Therapy, University of São Paulo, Ribeirão Preto, Brazil; 3 Department of Clinical Analyses, Toxicology and Food Sciences, University of São Paulo, Ribeirão Preto, Brazil; 4 Department of Morphology and Genetics, Federal University of São Paulo, São Paulo, Brazil; 5 Hematology, Medical School, University of São Paulo, São Paulo, Brazil; 6 Department of Internal Medicine, University of Pernambuco, Recife, Brazil; 7 Hematology Division, Federal University of Rio Grande do Sul, Porto Alegre, Brazil; 8 Hematology and Hemotherapy Center, University of Campinas, Campinas, Brazil; 9 Hematology Division, Federal University of Paraná, Curitiba, Brazil; 10 Hematology Division, Federal University of Minas Gerais, Belo Horizonte, Brazil; 11 Federal University of São Paulo, São Paulo, Brazil; 12 Cell Therapy Program, Princess Margaret Cancer Centre, Toronto, Canada; 13 Leukemia Service, Memorial Sloan-Kettering Cancer Center/Weill Cornell Medical College, New York, USA; 14 Department of Oncology, St. Jude Children’s Research Hospital, Memphis, USA; 15 Department of Medical and Molecular Genetics, King’s College London School of Medicine, London, UK; 16 Department of Hematology, Hemostasis, Oncology, and Stem Cell Transplantation, Hannover Medical School, Hannover, Germany; 17 Department of Hematology, Erasmus University Medical Center, Rotterdam, Netherlands; 18 Department of Biopathology, Tor Vergata University, Rome, Italy; 19 Santa Lucia Foundation, Rome, Italy; 20 Department of Hematology, Valencia University Medical School, Valencia, Spain; 21 CIBERONC, Instituto Carlos III, Madrid, Spain; 22 Department of Medicine, Brigham and Women’s Hospital, Harvard Medical School, Boston, USA; 23 LIM31, Hematology, Medical School, University of São Paulo, São Paulo, Brazil

**Keywords:** Apoptosis, Prognostic markers, Acute myeloid leukaemia

## Abstract

Non-T cell activation linker (NTAL) is a lipid raft-membrane protein expressed by normal and leukemic cells and involved in cell signaling. In acute promyelocytic leukemia (APL), NTAL depletion from lipid rafts decreases cell viability through regulation of the Akt/PI3K pathway. The role of NTAL in APL cell processes, and its association with clinical outcome, has not, however, been established. Here, we show that reduced levels of NTAL were associated with increased *all-trans* retinoic acid (ATRA)-induced differentiation, generation of reactive oxygen species, and mitochondrial dysfunction. Additionally, NTAL-knockdown (NTAL-KD) in APL cell lines led to activation of Ras, inhibition of Akt/mTOR pathways, and increased expression of autophagy markers, leading to an increased apoptosis rate following arsenic trioxide treatment. Furthermore, NTAL-KD in NB4 cells decreased the tumor burden in (NOD scid gamma) NSG mice, suggesting its implication in tumor growth. A retrospective analysis of *NTAL* expression in a cohort of patients treated with ATRA and anthracyclines, revealed that *NTAL* overexpression was associated with a high leukocyte count (P = 0.007) and was independently associated with shorter overall survival (Hazard Ratio: 3.6; 95% Confidence Interval: 1.17–11.28; *P* = 0.026). Taken together, our data highlights the importance of NTAL in APL cell survival and response to treatment.

## Introduction

Non-T cell activation linker (NTAL), or linker for activation of T cells (encoded by the *LAT2* gene), or linker for activation of B cells (LAB)^1,2^, is a single-pass type III lipid raft-membrane protein expressed by normal B-cells, plasma cells, NK cells, mast cells, and monocytes^3,4^. In mast and B-cells, NTAL mediates signaling of high-affinity IgE receptors, which are regulated by phosphorylation^5,6^. NTAL was initially described as a homolog to LAT (linker for activation of T cells), which participates in signalosome dynamics in T cells^7^. Similarly to LAT, NTAL possesses tyrosine-based activation motifs^8^, and interacts with signaling molecules, such as Grb2, Sos1, Gab1, and c-Cbl^5^. These findings reinforce the relevance of NTAL in important multicomponent complexes regulating downstream steps of signaling cascades.

*NTAL* is reported to be expressed in acute myeloid leukemia (AML) cells, but its expression varies significantly among the different subtypes of AML^9^. NTAL expression in primary AML blasts has already been found to be associated with myelomonocytic features^10^. NTAL protein levels are significantly decreased in a time-dependent manner in NB4 cells (an acute promyelocytic leukemia [APL] cell line) treated with *all-trans* retinoic acid (ATRA). Similarly, decreased NTAL expression has also been observed in other AML cell lines treated with drugs that induce differentiation^9,10^. In APL, NTAL depletion from lipid rafts in response to arsenic trioxide (ATO) decreases cell viability through regulation of the Akt/PI3K pathway^11^. However, the cellular processes in which NTAL is involved and the relevance to treatment response remain unexplored.

In the present study, we performed a knockdown (KD) of the *NTAL* gene and analyzed its effect on differentiation, apoptosis, autophagy, and mitochondrial function of APL cells (NB4 and NB4-R2), as models of a more genetically and clinically homogeneous AML cell line. NB4-R2 cells are a variant of the NB4 cells, with a mutation in the RARA portion (L900P) of the PML-RARA protein^12^ that leads to significantly reduced response to ATRA treatment. Moreover, we characterized changes in the phosphorylation of signaling proteins and evaluated the relevance of NTAL to ATRA or ATO treatment (the two primary drugs used to treat APL^13^ patients). Finally, we quantified *NTAL* transcript levels in samples from a patient cohort uniformly treated with ATRA and anthracyclines (International Consortium On Acute Promyelocytic Leukemia – IC-APL, 2006 study)^14^, and showed that *NTAL* overexpression was independently associated with shorter overall survival (OS). Taken together, our data highlights the importance of NTAL in APL cell survival and response to treatment.

## Results

### NTAL mediates ATRA-induced differentiation and NTAL knockdown decreases cell viability and proliferation

**Figure 1:**
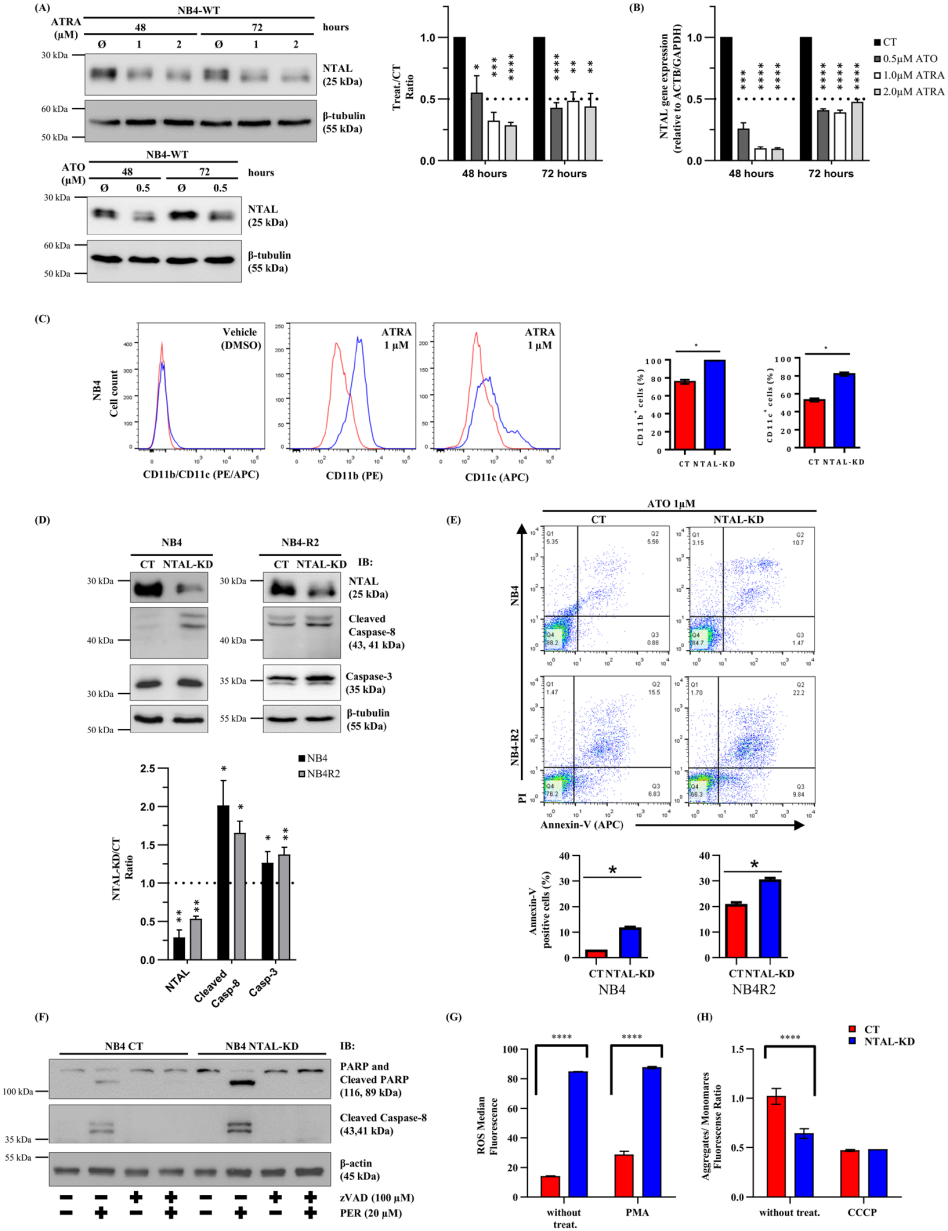
Non-T cell activation linker (NTAL)-knockdown (KD) increases all-trans retinoic acid (ATRA)-induced differentiation, apoptotic molecular markers and ROS activation. (**A**) Protein levels of NTAL after 48 h and 72 h of ATRA (1 or 2 µM), or arsenic trioxide (ATO) (0.5 µM) treatment in NB4 wild-type cells. Bar graphs show treatment to control ratio. Values are shown as the mean ± SEM, and (**B**) decreases NTAL mRNA expression levels (**C**) Representative flow cytometry analysis of CD11b and CD11c expression in NB4 cells (CT [control] and NTAL-KD) after 72 h of ATRA (1 µM) stimulation for differentiation. Bar graphs present the median of positive cells (percentage) analyzed by flow cytometry for cell lines transduced. (**D**) Effect of knockdown of the NTAL protein in NB4 and NB4-R2 cells (CT and NTAL-KD) on apoptotic markers (caspase-3 and caspase-8). Bar graphs show the NTAL-KD to CT ratio. Values are show as the mean ± SEM. (**E**) Effect of knockdown of the NTAL protein in NB4 and NB4-R2 cells (CT and NTAL-KD) on annexin-V^+^ cells at baseline, and following ATO-treatment (1 µM) for 24 h. (**F**) Analysis of apoptotic markers in NB4 (CT and NTAL-KD) cells pre-treated with 100 μM zVAD-fmk (zVAD) for 1 h, then treated with 20 μM perifosine for 6 h. (**G**) General ROS accumulation was evaluated by flow cytometry (2′,7′-dichlorofluorescein diacetate [H_2_DCFDA] fluorescence). For a positive control, NB4 cells were incubated with PMA (50 nM) to induce ROS accumulation through PKC activation, for 1 h prior to the analysis. (**H**) Δψ (mitochondrial membrane potential) was evaluated using the JC-1 aggregate/JC-1 monomer fluorescence ratio. Values are show as the mean ± SEM (*P < 0.05; **P < 0.01;***P < 0.001; ****P < 0.0001). NB4 and NB4-R2 cell lines were independently analyzed, CT vs. NTAL-KD.

To explore the molecular effects of NTAL on APL cells, we first evaluated the modulation of NTAL protein levels in NB4 cells treated with different concentrations of ATRA and ATO for 48 and 72 hours. As depicted in Fig. 1A, both drugs induced a reduction in NTAL protein levels in a dose-dependent manner. We also measured NTAL mRNA expression following ATRA and ATO treatment (Fig. 1B). To investigate NTAL function, NB4 and NB4-R2 (ATRA-resistant) cells were transduced with three different shRNA sequences. Cells transduced with sequence TNRC000128292 exhibited a higher level of NTAL inhibition compared to the control (CT – cells transduced with scrambled RNA) and was chosen for further functional assays (Supplementary Fig. S1A).

Following ATRA treatment, NTAL-knockdown (NTAL-KD) cells displayed a higher proportion of CD11b- and CD11c-positive cells in response to ATRA (NB4 NTAL-KD cells) treatment compared to the NB4-CT cells (Fig. 1C and Supplementary Fig. S1B,C).

To evaluate whether the decrease in NTAL is involved in regulating cell survival, caspase levels were measured at baseline in NTAL-KD cells and compared to the CT cells. NB4 NTAL-KD cells showed increased levels of caspase-3 and caspase-8 (Fig. 1D) resulting in increased numbers of apoptotic cells at baseline (i.e., spontaneous apoptosis), and following ATO-treatment (Fig. 1E). In addition, induction of apoptosis in response to the Akt inhibitor, perifosine, was assessed in NB4-CT and NB4-NTAL-KD cells, alone, or in combination with zVAD (an irreversible pan-caspase inhibitor). NB4-NTAL-KD cells exhibited higher sensitivity to perifosine (with increased cleavage of caspase-8 and PARP) in comparison with NB4-CT cells, while no differences were detected with perifosine in combination with zVAD (Fig. 1F). In addition, NTAL-KD cells showed increased reactive oxygen species (ROS) levels and loss of the mitochondrial membrane potential (Fig. 1G,H and Supplementary Fig. S1C).

### NTAL-knockdown modulates the AKT-mTOR and the MAPK-ERK-RAS signaling pathways

**Figure 2:**
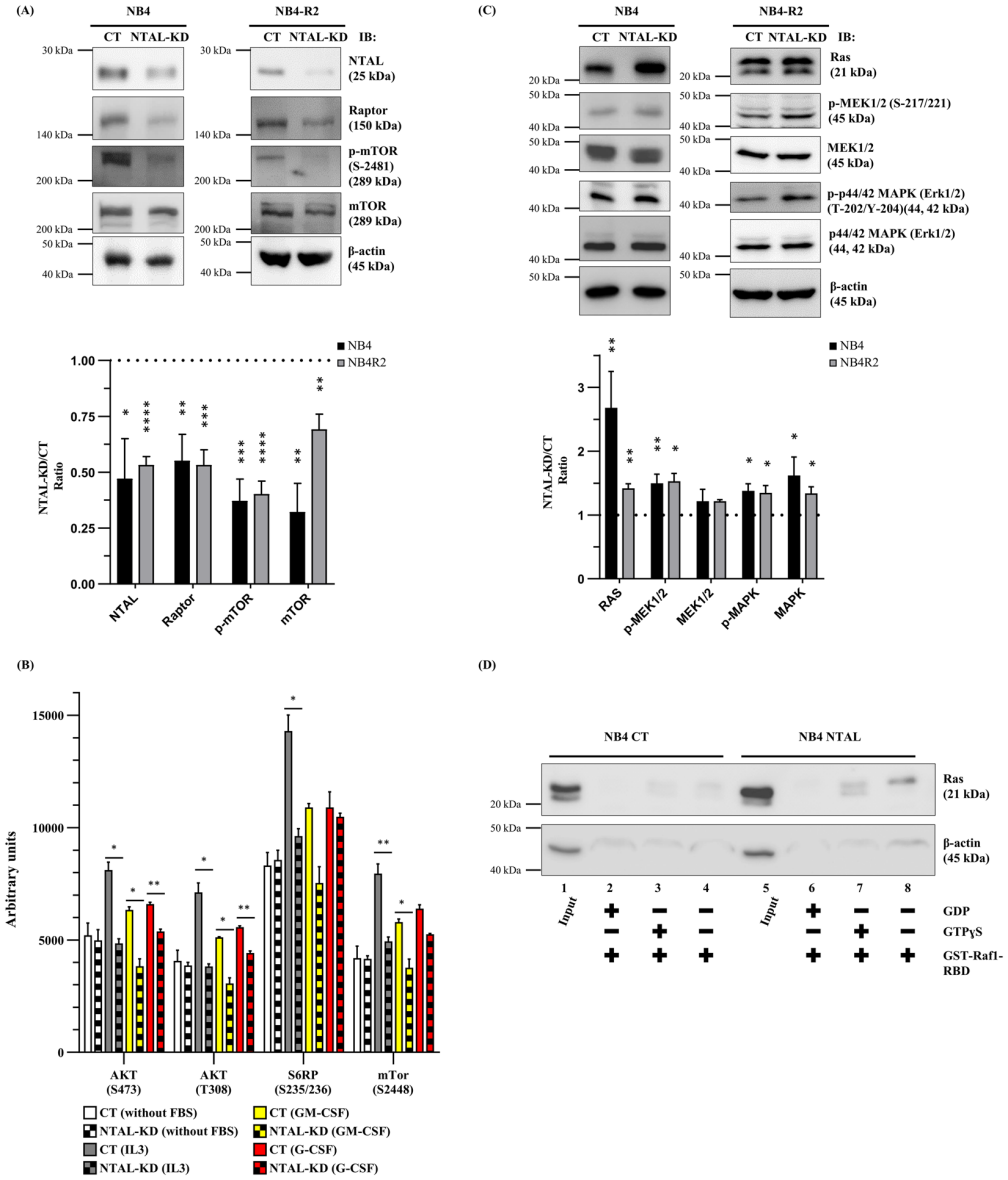
Non-T cell activation linker-knockdown (NTAL-KD) modulates the Akt/mTOR and Ras/MAPK pathways. (**A**) Western blotting analyses of the NTAL-knockdown effect in NB4 and NB4-R2 cells on proteins participating on the mTOR pathway. Bar graphs represent the NTAL-KD to control (CT) ratio. Values are show as the mean ± SEM. (**B**) Effect of NTAL protein on the activation of the Akt pathway after addition of myeloid growth factors (MGF). NB4 cells (CT or NTAL-KD) were cultured for 16–18 h in the absence of FBS, whereupon MGF (hr-IL-3 or hr-GM-CSF or hr-G-CSF or hr-SCF) was added. Aliquots were withdrawn after 15 min of each treatment and evaluated with the PathScan Intracellular Signaling Array Kit. Values were obtained using the combined signal of four individual spots. Values are show as the mean ± SEM. (**C**) Western blotting analyses of NTAL-knockdown effect in NB4 and NB4-R2 cells on proteins participating on MAPK pathway. NB4 and NB4-R2 cell lines were independently analyzed, CT vs. NTAL-KD. Bar graphs show the NTAL-KD to CT ratio. Values are shown as the mean ± SEM. (**D**) NB4 cells (CT or NTAL-KD) were treated with GTPγS (final concentration of 0.1 mM), or GDP (final concentration of 1 mM) to activate or inactivate RAS, respectively (Active Ras assay, Cell Signaling Technology Kit #8821). Cell lysates (500 µg) were incubated with GST-Raf1-RBD resin to pool-down active Ras. Western blotting of the pull-down eluted samples (lanes 2, 3, and 4 or 6, 7 and 8) was performed using a Ras mouse mAb. Cell lysate (20 µg, lane 1 or 5) was used as input control. Values are show as the mean ± SEM (*P < 0.05; **P < 0.01; ***P < 0.001; ****P < 0.0001). NB4 and NB4-R2 cell lines were independently analyzed, CT vs. NTAL-KD.

In APL cell lines, NTAL-KD cells exhibited decreased levels of Raptor, p-mTOR (Ser2481), and total mTOR compared with the CT cells (Fig. 2A). No significant difference in p-Akt (Ser473) and total Akt levels were detected in NB4-NTAL-KD cells (Supplementary Fig. S1D). NB4-NTAL-KD cells stimulated with myeloid growth factors (MGF) resulted in a hypophosphorylated state of Akt and downstream targets (Fig. 2B). The levels of Ras, p-MEK1/2 (Ser217/221), and p-p44/42 MAPK (ERK1/2-Thr202/204) were increased in APL NTAL-KD cells, with no significant differences in total MEK1/2 and p44/42 MAPK (ERK1/2) levels (Fig. 2C). Ras activation in the NB4-NTAL-KD cells was confirmed using the GST-Raf1-RBD fusion protein (Fig. 2D).

In NB4 cells, NTAL-KD increased the levels of PI3K-III, p-AMPKα (Thr172), AMPKα, Beclin-1, LC3-I/II, and ATG5, and decreased SQSTM1/p62 levels compared to CT, suggesting that NTAL-KD increased autophagy flux. In support of this, lysosome accumulation was higher in NB4-NTAL-KD cells at basal level and following treatment with drugs that regulate the autophagy process (Chloroquine and Rapamycin) compared to the CT cells. The ATG5 autophagy marker levels were increased in NB4 NTAL-KD cells at baseline, and further increased following the treatments described above. Compared to the CT cells, NB4 NTAL-KD cells presented lower levels of LC3-II following treatment with chloroquine and rapamycin, reinforcing the theory that NTAL participates in the regulation of the autophagic flux (Supplementary Fig. S2A–D). A similar strategy was used to compare the induction of autophagy in NB4-NTAL-KD and NB4-CT cells using 3-Methyladenine (3-MA) as an inhibitor. Regardless of 3-MA treatment, NB4-NTAL-KD cells demonstrated higher autophagic flux than NB4-CT cells (Supplementary Fig. S3A). Taken together, these data suggest that NTAL is involved in the regulation of apoptosis and autophagic flux in APL cells.

We also used transmission electron microscopy to assess morphological alterations promoted by NTAL-KD at the ultrastructural level. NB4-NTAL-KD cells demonstrated a decrease in cytoplasmic vacuolization with the presence of mitochondrial degeneration (Supplementary Fig. S3B).

### NTAL-knockdown resulted in smaller tumors in a xenograft model

**Figure 3:**
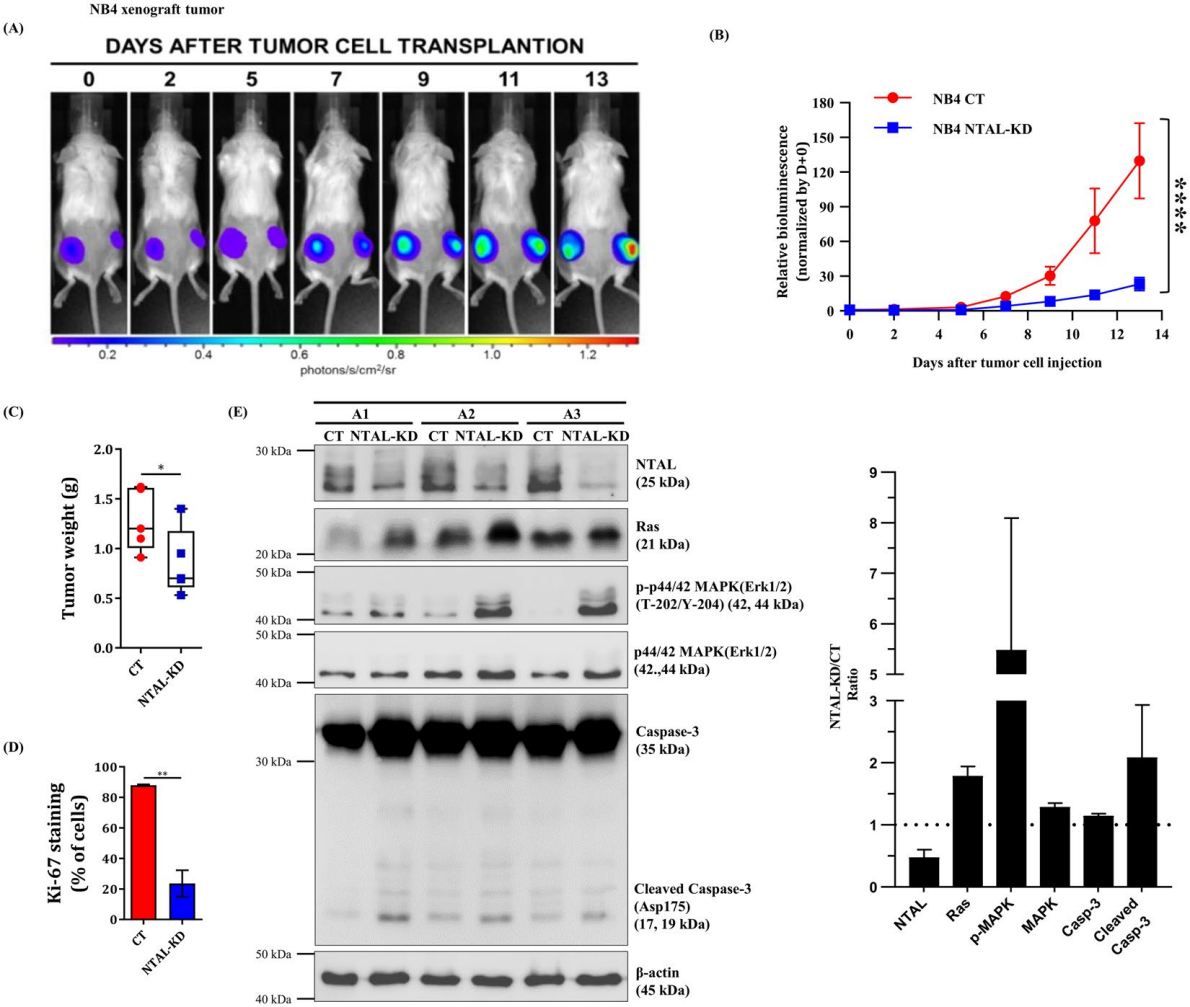
Characterization of the xenograft model of the NB4 cell line in NSG mice. Female 12-week-old NSG mice were injected subcutaneously into the left thigh with 1 × 10^6^ non-T cell activation linker**-**knockdown **(**NTAL-KD) cells, and received an equal number of control (CT) cells in the right thigh subcutaneously. (**A**,**B**) Relationship between bioluminescence and size of the tumor mass. The bioluminescent signal intensity was measured 2, 5, 7, 9, 11, and 13 days after injection of CT tumor cells (right flank) and NTAL-KD cells (left flank) expressing GFP-LUC. Images were acquired with IVIS Lumina representing the location of the cells and the tumor size. (**C**) After 2 weeks, the animals were euthanized and the tumors excised, weighed and processed. A graph of tumor masses derived from NB4 (CT and NTAL-KD) after two weeks: NB4: CT (1.286 g ± 0.3137) and NTAL-KD (0.8540 g ± 0.3402) (n = 5, *P* = *0.0143*). (**D**) Immunohistochemical detection quantitative analysis of Ki67 proliferation marker. Data are reported as means and standard deviations. (**E**) Western blot analysis of proteins NTAL, total Ras, p-p44/42 MAPK (ERK1/2-Thr202/204), total p44/42 MAPK (ERK1/2) and caspase-3 tumors originating from NB4 (CT and NTAL-KD) (animals A1 = 1, A2 = 2 and A3 = 3). Bar graphs show the NTAL-KD to CT ratio from the three animals analyzed. Values are show as the mean ± SEM.

After identifying the participation of NTAL in proliferation and apoptosis, we analyzed markers of these processes in the engrafted tumors obtained from a xenograft model. Inoculation of NB4-NTAL-KD cells resulted in significantly smaller tumors and with lower Ki67-positive labeling indexes (Fig. 3A–D and Supplementary Fig. S4). Western blot analyses showed the same pattern of signaling proteins in NTAL-KD engrafted tumor models as seen in the *in vitro* studies (Fig. 3E). The levels of total Ras, p-p44/42 MAPK (ERK1/2-Thr202/204) and cleaved caspase-3 increased, while total and p44/42 MAPK (ERK1/2) protein remained similar in the NB4-NTAL-KD engrafted tumors samples when compared to that seen in the NB4-CT engrafted tumors samples.

### High *NTAL* transcript levels may predict lower overall survival in APL patients

**Figure 4:**
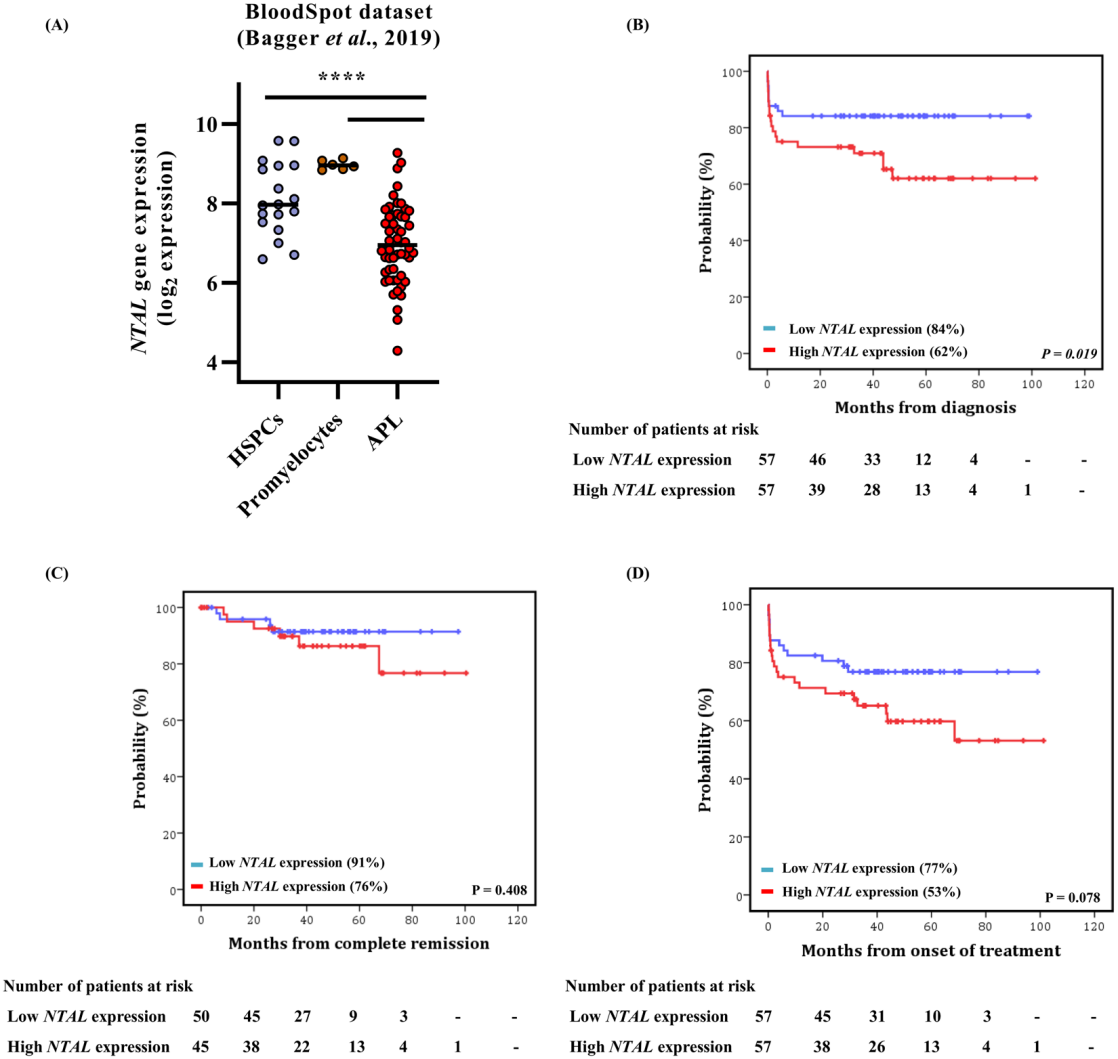
Differential gene expression of non-T cell activation linker *(NTAL)* in healthy bone marrow hematopoietic stem-progenitor cells (HSPCs), promyelocytes, and acute promyelocytic leukemia (APL). (**A**) *NTAL* transcript levels in samples from patients with APL and comparative controls identified in the *BloodSpot* databank, which was used as a validation cohort. *NTAL* transcript levels were compared between newly diagnosed APL patients and healthy donors cells, including HSPCs and purified promyelocytes. (**B**) The probability of overall survival (OS) (**C**), disease-free survival (DFS) (**F**), and event-free survival (EFS) (**D**), in patients with APL and relative to *NTAL* expression (International Consortium on Acute Leukemia – ICAL2006 cohort). The horizontal bars represent the median value of *NTAL* expression. NTAL expression was lower in APL samples as determined by the Kruskal-Wallis test followed by the Dunn post-hoc test.

Using public a databank (*BloodSpot*) we compared the values of *NTAL* expression in samples from newly diagnosed APL patients (n = 54) and healthy volunteers (n = 6). *NTAL* expression was significantly lower in APL bone marrow (BM) samples compared with the Hematopoietic Stem- and progenitor-cells and promyelocytes from healthy volunteers (*P* < 0.01; Fig. 4A).

We dichotomized patients according to the median value of *NTAL* expression and compared the groups of APL patients with low and high *NTAL* expression. Baseline characteristics were similar in the two groups (Table 1), except for higher white blood cell (WBC) counts and a higher frequency of patients with hyperleukocytosis (defined as patients with equal or more than 10 × 10^9^ cells/L) in the high *NTAL* group (P = 0.007). With a median follow-up of 76 months (1–101 months), the estimated 5-year overall survival (OS) rate was 73% (95% confidence interval, CI: 63–80%) in the entire cohort. Overall, 95/114 (83%) of APL patients achieved complete hematological remission (CHR). Of the 19 patients (17%) who failed to achieve CHR, 15 (79%) experienced early mortality (i.e., death within 30 days after diagnosis). *NTAL* expression had no impact on CHR achievement (*P* = 0.315), or on early mortality (P = 0.235). In contrast, patients with a high *NTAL* expression had a lower 5-year OS rate (62%, 95% CI: 46–74%) compared to patients with a low *NTAL* expression (84%, 95% CI: 71–91%) (hazard ratio, HR: 2.26, 95% CI: 1.02–5.01, *P* = 0.019; Fig. 4B). These findings were confirmed by a multivariate analysis, demonstrating that *NTAL* overexpression was independently associated with a shorter OS (HR: 3.6, 95% CI: 1.17–11.28, *P* = 0.026) (Table 2), with modifiers of treatment outcome: age, gender, WBC, and albumin levels^13^. *NTAL* expression showed no association with the disease-free survival and event-free survival rates (Fig. 4C,D).

**Table 1:** Baseline characteristics of APL patients according to NTAL expression levels.

Characteristic	All patients		*NTAL* expression				*P* value
			Low expression		High expression		
	No.	%	No.	%	No.	%	
**Gender**							0.990
Female	57	50	28	49.1	29	50.9	
Male	57	40	29	40.9	28	49.1	
Age, median	35.7		36.4		34.8		0.821
(range)	(18.9, 65.4)		(19, 65.4)		(18.9,65.4)		
**ECOG performance status**							0.097
0	61	57.5	38	66.7	23	46.9	
1	21	19.8	11	19.3	10	20.4	
2	13	12.3	6	10.5	7	14.3	
≥3	10	9.4	2	3.5	8	16.3	
Unknown	1	0.9	—	—	1	2	
**Relapse-risk group**							0.220
Low risk	18	15.8	11	19.3	7	12.3	
Intermediate risk	45	39.5	25	43.9	20	35.1	
High risk	51	44.7	21	36.8	30	52.6	
***PML*** **breakpoint**							0.386
BCR1	58	61.1	35	67.3	23	53.5	
BCR2	2	2.1	1	1.9	1	2.3	
BCR3	35	36.8	16	30.8	1319	44.2	
Unknown	19	—	5	—	14	—	
**Leukocyte counts, ×10**^**9**^**/L**							0.007
<5	51	45	25	44	26	46	
5–10	12	10	11	19	1	1	
>10	51	45	21	37	30	53	
Platelet counts (×10^9^/L), median	26		27		24		0.408
(range)	(4, 128)		(4, 128)		(4, 102)		
Hemoglobin (g/dL), (median,	8.8		8.4		9		0.167
range)	(3.4, 21.8)		(3.4, 14.1)		(4.7, 21.8)		
Creatinine (mg/dL), (median,	0.8		0.8		0.8		0.970
range)	(0.42, 2.8)		(0.5, 2.2)		(0.42, 2.8)		
Uric acid (mg/dL), (median,	3.8		3.8		3.8		0.735
range)	(1.1, 9)		(1.1, 9)		(1.3, 7.4)		
Fibrinogen (mg/dL), (median,	161		157		165		0.967
range)	(10, 898)		(10, 605)		(18, 898)		
Albumin, g/dL, (median,	3.9		4		3.9		0.097
range)	(2.2, 5.3)		(2.2, 5.3)		(2.2, 4.9)		

Abbreviations: WBC, white blood cells.

**Table 2:** Multivariable Cox model for overall survival.

All patients, No. (%): 114 (100)	Overall survival			
	HR	95% CI		*P*-value
*NTAL* expression: high versus low	3.6	1.17	11.28	0.026
Gender: male *versus* female	0.79	0.28	2.27	0.675
Age: continuous variable	1.02	0.99	1.05	0.090
WBC (×10^9^/L): continuous variable	1.01	1.01	1.03	<0.001
Albumin (g/dL): continuous variable	0.5	0.23	1.05	0.069

Note: Hazard ratio (HR) >1 or <1 indicates an increased or decreased risk, respectively, of an event for the first category listed.

### Internal validation data

The final prediction model was internally validated using a bootstrap resampling procedure with 10,000 repetitions from the original database to assess model bias. Internal validation resulted in an Area Under the Curve (0.641, 95% CI: 0.51 to 0.75) very similar to that described for the original data. The bootstrap results are depicted in Table 3. Briefly, for Overall Survival (OS) and Event Free-Survival (EFS) at different time points, the procedure yielded a mean and 95% CI virtually identical to its original match. Also, for all comparisons, the pairwise hypothesis testing showed significance (*P* < 0.0001) for the difference across the distributions means.

**Table 3:** Summary outcomes of patients according to the *NTAL* expression considering the original data and the bootstrap resampling.

Clinical endpoints	1-year, % of mean (95% CI)			2-year, % of mean (95% CI)			5-year, % of mean (95% CI)		
	Low *NTAL*	High *NTAL*	*P*-value	Low *NTAL*	High *NTAL*	*P*-value	Low *NTAL*	High *NTAL*	*P*-value
**Overall survival**			<0.001*			<0.001*			<0.001*
Original data	84 (72 to 92)	73 (59 to 83)		84 (72 to 93)	73 (59 to 83)		84 (72 to 91)	62 (46 to 74)	
Bootstrap resampling	84 (73 to 93)	73 (60 to 84)		84 (73 to 93)	73 (60 to 84)		84 (73 to 93)	62 (47 to 75)	
**Event-free survival**			<0.001*			<0.001*			<0.001*
Original data	82 (70 to 90)	71 (57 to 81)		81 (68 to 89)	69 (56 to 80)		77 (63 to 87)	60 (45 to 72)	
Bootstrap resampling	82 (70 to 91)	71 (58 to 82)		81 (69 to 90)	69 (56 to 81)		77 (64 to 87)	60 (46 to 73)	

*Welch two samples t-test.

Abbreviation: CI: confidence interval.

## Discussion

In the present study, we show that the knockdown of NTAL reduced mTOR activation and downstream targets, and increased levels of Ras-MAPK-ERK in NB4 cells. In addition, we observed increased expression of autophagic flux and apoptosis markers in NTAL-KD cells, at baseline and following ATO-treatment. These results reinforce the importance of NTAL in cell survival, and are in agreement with our previous results showing that NTAL depletion from lipid rafts decreases cell viability through regulation of the Akt/PI3K pathway^11^. We also identified signals of mitochondrial degradation in NB4-NTAL-KD cells. ATO-induced ROS in APL cells have been shown to originate from NADPH oxidase through the upregulation of virtually all components of this mitochondrial membrane-associated enzyme complex^15^.

NTAL was also involved in the granulocytic differentiation of APL blasts treated with ATRA. The inhibition of mTOR^16^ and activation of Ras-Raf-MAPK-ERK^17,18^ pathways in the regulation of myeloid differentiation may explain these findings. ERK and JNK activation can stimulate the autophagic removal of mitochondria during oxidative stress^19^. *In vivo*, the inoculation of NB4-NTAL-KD cells resulted in lower tumor growth rates in immunodeficient mice compared to controls.

*NTAL* expression was significantly reduced in APL blasts when compared to normal bone marrow mononuclear cells. In a cohort of patients treated with ATRA and anthracycline-based chemotherapy, high *NTAL* levels were associated with high WBC counts, and this may explain the independent association with lower OS, without impacting on CHR rate^13^. Despite the progress in APL treatment, 10–15% of patients relapse after treatment with ATRA plus chemotherapy, and frequently present with ATRA resistance^20^. Arsenic can circumvent this scenario, but its cost and health surveillance agencies politics remain a significant barrier for many low- and middle- income countries. In the context of the ATRA plus chemotherapy, we have described other molecular markers that carry prognostic information^21–23^, suggesting that heterogeneity in APL outcomes may be higher than expected. Previously, we showed that NTAL is a primary protein involved during 10-(octyloxy) decyl-2-(trimethylammonium) ethyl phosphate (ODPC) treatment in different leukemic cell lines^11^, not only for APL but for AML as well. Here, we looked at the role of the NTAL gene and protein expression in APL only, as a homogenous model of AML. The downregulation of NTAL led to decreased activation of Akt/PI3K and downstream pathways, impaired cell proliferation and survival, and an increased sensitivity to the primary drugs used in the clinical setting at equivalent doses applied during patient treatment. Furthermore, the increased doses of ATRA showed that the downregulation of NTAL mRNA and protein levels were both time and dose-dependent. We demonstrated that increased *NTAL* levels were associated with decreased OS, regardless of other risk variables. Interestingly, NTAL expression was decreased in APL samples compared to healthy controls. This could argue for the fact that NTAL is not associated with malignant transformation per se, but seems to exert its functional effects on APL blasts cells, orchestrating several biological processes that dictate a more aggressive biology. Although NTAL-KD cells exhibited a high sensitivity to ATO, recent data have shown that some prognostic markers established for conventional ATRA and chemotherapy regimens could not be reproduced in the case of treatment with ATO^24^. The potential role of Akt-inhibitor treatment, which results in NTAL degradation^11^ in ATRA and ATO refractory APL cases, remains to be evaluated.

In conclusion, our results showed that reducing NTAL levels led to anti-leukemic activity in APL cells *in vitro* and *in vivo*. Our results support the notion that NTAL is involved in the cross talk between proliferation, differentiation, and autophagy in APL cells, and that high *NTAL* expression its associated with decreased OS in APL patients.

## Methods

All methods were carried out in accordance with the approved guidelines.

### List of materials

zVAD-fmk (627610) was purchased from Calbiochem (Gibbstown, NJ, USA). Chloroquine (#14774) and LysoTracker Green DND-26 (#8783) were purchased from Cell Signaling (Danvers, MA, USA). 3-Methyladenine (3-MA) (M9281), Bafilomycin-A1 (B1793), Phorbol-12-myristate-13-acetate (PMA) (P8139), Rapamycin (R0395), All-trans retinoic acid (ATRA) (R2625) and arsenic trioxide (A1010) were purchased from Sigma (St Louis, MO, USA). Perifosine (S1037) was purchased from Selleck Chemicals (Houston, TX, USA).

### Cell lines

The two APL cell lines, NB4 (ATRA-sensitive) and NB4-R2 (ATRA-resistant), were donated by Dr. Pier Paolo Pandolfi (Harvard Medical School), and maintained as recommended by DSMZ (Braunschweig, Germany), in RPMI 1640 (Gibco, USA) supplemented with 10% fetal bovine serum (SFB) (Gibco), L-glutamine (2 mM), and penicillin/streptomycin (Invitrogen). Mycoplasma contamination was routinely tested (once per month). The cell lines were authenticated by short tandem repeat analysis (last authentication – November 2018).

### Granulocytic differentiation induction

Cell suspensions containing 2 × 10^5^ of transduced cells were incubated in RPMI medium containing 10% FBS for 72 h in presence of ATRA (1 µM) for NB4 and NB4-R2 cells. The differentiation rate was determined by immunophenotyping using the percentage (%) and the MFI of CD11b- and CD11c-positive cells (BD Biosciences, San Jose, CA, USA) as maturity marker. Experiments were acquired in a FACSCalibur flow cytometer (Becton-Dickinson) and analyzed using FlowJo software (Treestar, Inc).

### Lentiviral production

Lentiviral particles were produced in HEK293FT cells using the ViraPower Lentiviral Expression System (LifeTechnologies). Briefly, 1 µg of the plasmid of interest and 3 µg of packing plasmids were mixed with 12 µl of Lipofectamine-2000 (LifeTechnologies) in 2 ml of DMEM medium (antibiotics- and FBS-free), then incubated with HEK293FT cells overnight. The medium was replaced with complete DMEM medium. After 48 h of transfection, the medium was collected and centrifuged at 5 000 × g for 15 min at 4 °C. Aliquots were immediately used for lentiviral transduction or stored at −80 °C.

### Cell transfection

Cell transfection was performed with lentiviral particles containing plasmid-DNA for GFP (green fluorescent protein) and firefly luciferase (pGF_CMV_dscGFP_LUC plasmid) or MISSION TurboGFP Control (pLKO.1) (Sigma) or MISSION shRNA NTAL (SHCLNV-NM_014146). The MISSION-shRNA sequence selection was performed using the Broad Institute RNAi consortium data bank^25^. For transfection, 6.4 × 10^5^ cells were plated with medium containing viral particles and 8 µg/ml polybrene (Sigma) overnight. Cells were washed twice and resuspended in complete RPMI medium. After 48–72 h GFP-positive cells were sorted by flow cytometry (JSAN, BayBiosciences, Kobe, Japan) until the cell population showed more than 99% GFP expression. MISSION TurboGFP Control (pLKO.1) or MISSION shRNA Plasmid NTAL were selected with puromycin (Sigma), 0.5 µg/ml NB4 or NB4-R2 cells, for 3–5 passages (~10 days).

### Protein extraction and quantification

Cells were washed with PBS by centrifugation, disrupted in lyses buffer (20 mM Tris-HCl (pH 7.5), 150 mM NaCl, 1 mM Na_2_EDTA, 1 mM EGTA, 1% Triton X-100, 2.5 mM sodium pyrophosphate, 1 mM β-glycerophosphate, 1 mM Na_3_VO_4_ and 1 µg/ml leupeptin) and homogenized in a D-130 tissue homogenizer (Biosystems, Brazil) at 15 000 rpm on ice and centrifuged at 20 000 × g for 30 min at 4 °C. The protein concentration was determined by the Bradford method (Bio-Rad, Hercules, CA).

### Western blotting

Samples were separated by SDS–PAGE (7.5 or 10% or 12%) and electro transferred to PVDF membranes (GE Lifesciences, Pittsburgh, PA, USA). The membranes were blocked with wash buffer (25 mM Tris-HCl, pH 7.5, 0.5 M NaCl and 0.1% Tween-20) containing 5% non-fat dry milk and incubated with a primary antibody following manufacturer’s instructions (see Supplementary Table S1). The following secondary antibodies were used: horseradish peroxidase-conjugated goat anti-rabbit IgG (#7074) or horse anti-mouse IgG secondary antibody (#7076) or streptavidin-HRP conjugate (#3999) (Cell Signaling). Targets were visualized with ECL Western blotting Detection Reagents (GE Lifesciences) using a CCD-Camera (Image Quant LAS 4000 mini, Uppsala, Sweden), and images were acquired using precision and/or increment mode. Densitometric analysis was performed using the ImageJ software^26^, and bands were normalized to constitutive proteins (β-actin, β-tubulin or GAPDH). Western blots were statistically analyzed by two-tailed unpaired t test.

### Apoptosis assay

NB4 and NB4-R2 cells were seeded in 24-well plates and treated with ATO (1 µM) and vehicle (NaOH) for 24 h. Cells were then washed twice with ice cold PBS and resuspended in binding buffer containing 1 µg/ml propoidium iodide (PI) and 1 µg/ml APC-labeled annexin V (BioLegend, San Diego, CA, USA). All specimens were acquired by flow cytometry (FACSCalibur; Becton-Dickison) after incubation for 15 min at room temperature and analyzed using FlowJo software (Treestar, Inc., San Carlos, CA, USA).

### ROS detection by fluorescence and mitochondrial membrane potential (Δψ) assay

Production of ROS and Mitochondrial membrane potential (Δψ) were evaluated using the intracellular fluorogenic reagent CM-H_2_DCFDA (C6827, ThermoFisher Scientific) and JC-1 (5,5′,6,6′-tetrachloro-1,1′,3,3′-tetraethylbenzimidazolcarbocyanine iodide) (T3168, Molecular Probes, Eugene, OR, USA), respectively, according to the manufacturer’s instructions. The APL cell lines (CT and NTAL-KD) cells were incubated with 5 μM of CM-H_2_DCFDA for 1 h prior to the analysis, or 1 µM JC-1 for 20 min at 37 °C in a 5% CO_2_ atmosphere for 20 min. Cells were then washed and resuspended in PBS. The ROS and JC-1 were detected on a FACSCalibur cytometer (Becton-Dickinson) in the FL1/FL2 channel, respectively, and analyzed using FlowJo software (Treestar, Inc).

### Akt hypo-phosphorylation and phosphorylation

NB4 cells (CT and NTAL-KD) were maintained in culture in serum-free medium overnight (16–18 h). Cells were then stimulated with myeloid growth factors (10 ng/ml hr-IL-3 or 10 ng/ml hr-GM-CSF, 10 ng/ml hr-G-CSF, 50 ng/ml hr-SCF) (PeproTech, Mexico City, Mexico). Aliquots were removed after 15 min of stimulation and assayed by antibody array.

### PathScan intracellular signaling array kit

The PathScan Intracellular signaling array kit containing fixed antibodies against phosphorylated proteins by the chemiluminescent sandwich ELISA format was used according to manufacturer’s instructions (#7323, Cell Signaling). Images were analyzed with LI-COR Image Studio v4.0 analysis software by loading the image as a gray scale picture. Each protein array dot was selected manually, and an average intensity was calculated for each protein. Normalization within one stimulation experiment was done by subtracting the intensity of the negative control dot from each value. For comparison of different conditions, sets were normalized so that the positive controls had nearly equal intensities.

### Active ras detection assay

Active Ras protein in NB4 (CT and NTAL-KD) cells was evaluated using (#8821, Cell Signaling) according to manufacturer’s instructions. NB4 (CT or NTAL-KD) cells lysates (500 µg), treated with GTPγS (for a final concentration of 0.1 mM) or GDP (for a final concentration of 1 mM) to respectively activate or inactivate RAS, and non-treated were incubated with GST-Raf1-RBD and gluthatione-agarose resin to pool-down active Ras. Input and pull-down samples were separated by SDS-PAGE and western blotting analysis against Ras.

### Transmission electron microscopy

The NB4 (CT and NTAL-KD) cells were treated overnight or not with 10 µM chloroquine. The cells were fixed in 2% glutaraldehyde in 0.1 M sodium phosphate buffer at pH 7.4 (PB) for 2 h. The specimens were post-fixed in PB buffer containing 1% OsO_4_ for 2 h, and dehydrated in an ethanol series (30%, 50%, 70%, 80%, 95%, and 100%), for 10 min each, followed by 2 times with propylene oxide, for 5 min each. They were then infiltrated with propylene oxide and epoxy resin (V/V = 1:1), embedded with EPON 812 epoxy resin, DDSA, DMP-30, and MNA resin, and then aggregated for 24–48 h at 60 °C. Ultra-thin sections (60–70 nm) were cut with a diamond knife and stained with uranyl acetate and lead citrate. Sections were examined with a TEM (Jeol, Jem 100cx, Tokyo, Japan). Pictures were taken and converted to digital files (Hamamatsu, ORCA-HR Amtv542, Hamamatsu City, Japan).

### Animal xenograft studies

All animal studies were approved by the Animal Ethics Committee of the Faculdade de Medicina de Ribeirão Preto (FMRP) - University of São Paulo (USP) (protocol #135/2014). All methods were carried out in accordance with the approved guidelines and to IACUC guidelines.

### Tumor engraftment in murine model

Female 12-week-old NSG mice were maintained receiving NUVITAL (autoclavable rodents’ pellets) and water (autoclaved) ad libitum, under a 12/12 light/dark cycle, at 23 °C environmental temperature and 55% relative humidity. Mice were injected subcutaneously with 1 × 10^6^ CT cells into the right lateral thigh or with NTAL-KD cells into the left lateral thigh. After 2 weeks, all the animals were euthanized and the tumors excised, weighed and processed for further analysis. No randomization or exclusion criteria were used for the animal studies. Animal procedures complied with the guidelines on animal experimentation for the protection and humane use of laboratory animals. All procedures used were approved by the Ethics Committee of the Faculdade de Medicina de Ribeirão Preto (FMRP) - USP (protocol #135/2014).

### Bioluminescent imaging

Bioluminescent imaging was assessed by injecting mice intraperitoneally with 150 mg/kg D-luciferin (Perkin Elmer, Waltham, MA, USA) and placed in the Lumina *in Vivo* Imaging System (Perkin Elmer) chamber under continuous exposure to 1.5% isoflurane (Abbot Laboratories, Brazil) every two days after post engraftment. The bioluminescence intensity is reported as photon flux (photons/s).

### Histology and Immunohistochemistry

Hematoxylin and eosin staining and immunohistochemistry (IHC) analysis against NTAL and Ki67 were performed on 10% formalin-fixed, paraffin-embedded tissue sections.

### Patients, treatment protocol, ethics approval, and consent to participate

To validate our gene expression data, we analyze the *NTAL* transcript levels (Probe, #211768_at) in normal bone marrow mononuclear cells (BMMC), purified promyelocytes and CD34^+^ cells and newly diagnosed APL samples available at the BloodSpot databank (http://servers.binf.ku.dk/bloodspot/). Details about the cohort design and gene expression evaluation are described published elsewhere^27^.

A total of 114 consecutive patients with newly diagnosed APL who were enrolled in the IC-APL study were included. Details regarding diagnosis, classification, and treatment protocol are published elsewhere^14^.

All procedures were approved by the Ethics Committee of the Faculdade de Medicina de Ribeirão Preto – USP, and by the National Commission of Ethics in Research, National Health Council, Ministry of Health (Conep) (registry: 12920; process number: 13496/2005; CAAE: 155.0.004.000-05). Informed consent was obtained from all patients and approved by the Research Ethics Board, as described in the Ethics approval and consent to participate section. All methods were carried out in accordance with the approved guidelines and the Declaration of Helsinki.

### Gene expression profile of *NTAL*

All samples used for gene expression analyses were obtained at diagnosis from bone marrow aspirates and were processed according to standard techniques. Following total RNA extraction, real-time quantitative polymerase chain reaction (RQ-PCR) assays with sample-derived cDNA were performed in duplicate on MicroAmp optical 96-well plates using a 7500 Real-Time PCR System (Applied BioSystems, Foster City, CA, USA) with the *GAPDH* and *ACTB* Standard Kit as endogenous controls. *NTAL* gene expression was determined by real-time reverse transcriptase polymerase chain reaction using TaqMan Gene Expression Assay (Hs00247916_m1, Applied BioSystems), following the manufacturer’s instructions. The gene expression of *NTAL* was calculated relative to a reference cDNA (NB4 cell line, a human acute promyelocytic leukemia cell line, positive for PML/RARA fusion gene) and set at 1. Of importance, the same reference cDNA was used as an internal control throughout all experiments to ensure that the results would be fully comparable among experiments. The gene expression values of *NTAL* were calculated as relative quantification using the ∆Ct method and expressing the results as 2-ΔΔCt, in which ΔΔCt = ΔCt_patients_ − ΔCt_NB4 cell line_.

### Bootstrap with replacement analysis

In order to cross-validate our findings, we have performed a non-parametric bootstrap procedure with 10 000 resamplings of the original cohort allowing replacement^28^. The function calculated a given survival (e.g., overall survival or disease-free survival) in three different time points (12-months, 2-years, and 5-years) for both categories (low and high *NTAL* expression), and it also estimated their respective 95% confidence interval computing the bias-corrected and accelerated (BCa) bootstrap interval. We also had tested the hypothesis that the mean of both bootstrap sampling distributions was different using either a Welch two sample t-test when the distributions were normal in a QQ plot inspection or using a Wilcoxon rank-sum test when not normal.

### Statistical analysis

Patient baseline characteristics were reported descriptively. With the use of survival ROC curve analysis^29^, we dichotomized patients according to the median value of *NTAL* expression (low expression, <0.17; high expression ≥0.17). All *P* values were two-sided with a significance level of 0.05. In order to cross-validate our findings, we performed a non-parametric bootstrap procedure with 10,000 resamplings of the original cohort allowing replacement^30,31^.

Patient baseline characteristics were reported descriptively. Fisher’s exact test or Chi-square test, as appropriate, was used to compare categorical variables, and Kruskal-Wallis test was used to compare continuous variables. According to survival receiver operating characteristic (ROC) curve analysis (area under the curve, AUC: 0.641, 95% CI: 0.521–0.762), the median value of NTAL expression (median value: 0.17, range: 0.039–3.91; sensitivity: 69%; 1-specificity: 44%) was used to dichotomize patients into two groups (i.e., low expression, <0.17; high expression, ≥0.17). Clinical endpoints were previously described.

Univariable and multivariable proportional hazards regression analysis associated with interactions between the variables were performed for potential prognostic factors for overall survival (OS), event-free survival (EFS) and disease-free survival (DFS). OS was defined as the time from diagnosis to death from any cause; those alive or lost to follow-up were censored at the date last known alive. Early mortality was defined as death occurring within 30 days from diagnosis. For patients who achieved CR, DFS was defined as the time from CR achievement to the first adverse event: relapse, development of secondary malignancy, or death from any cause, whichever occurred first. EFS was defined as the time from the initiation of induction therapy to disease relapse, development of secondary malignancy, or death from any cause, whichever occurred first. Patients who were alive without disease relapse or secondary malignancy were censored at the time they were last seen alive and disease-free. Potential prognostic factors examined in multivariable regression analysis were age at diagnosis, gender, white blood cell (WBC) counts, platelet counts, hemoglobin levels, coagulopathy, FAB classification (M3 or M3v), PML breakpoint, creatinine, albumin, uric acid, and fibrinogen and *FLT3*-ITD mutation (with allele ratio evaluation). Linearity assumption for all continuous variables was examined using restricted cubic spline estimates of the relationship between the continuous variable and log relative hazard/risk. All P values were two sided with a significance level of 0.05.

All calculations were performed using Stata Statistic/Data Analysis version 14.1 (Stata Corporation, USA), statistical package for the social sciences (SPSS) 19.0 and R 3.3.2 (The CRAN project, www.r-project.org) software.

## Supplementary information

Supplementary Data.

## Data Availability

The data used and analyzed during this study are available from the corresponding author on request.
