# Supplementary material for: NTAL is associated with treatment outcome, cell proliferation and differentiation in acute promyelocytic leukemia
Source: Sci Rep. 2020 Jun 25;10:10315. doi: 10.1038/s41598-020-66223-2 (PMC7316767; doi:10.1038/s41598-020-66223-2)

NTAL is associated with treatment outcome, cell proliferation and differentiation in acute promyelocytic leukemia.

Carolina Hassibe Thomé[1,2*], Germano Aguiar Ferreira[1,2*], Diego Antonio Pereira-Martins[2*], Guilherme Augusto dos Santos[2], César Alexander Ortiz[2], Lucas Eduardo Botelho de Souza[2], Lays Martins Sobral[3], Cleide Lúcia Araújo Silva[2], Priscila Santos Scheucher[2], Cristiane Damas Gil[4], Andréia Machado Leopoldino[3], Douglas R. A. Silveira[5], Juan L. Coelho-Silva[2], Fabíola Traina[2], Luisa C. Koury[2], Raul A. M. Melo[6], Rosane Bittencourt[7], Katia Pagnano[8], Ricardo Pasquini[9], Elenaide C. Nunes[9], Evandro M. Fagundes[10], Ana Beatriz F. Gloria[11], Fábio Kerbauy[11], Maria de Lourdes Chauffaille[12], Armand Keating[13], Martin S. Tallman[14], Raul C. Ribeiro[15], Richard Dillon[16], Arnold Ganser[17], Bob Löwenberg[18], Peter Valk[18], Francesco Lo-Coco[19,20], Miguel A. Sanz[21,22], Nancy Berliner[23], Vitor Marcel Faça[1,2#] and Eduardo M. Rego[2,5#]

[* and #] These authors contributed equally to this work.

.

SUPPLEMENTARY DATA

Supplementary Table S1. Sources and properties of antibodies used in the work.

	Antibody	Company	Catalog Number	Source	P/M	Mass (kDa)	Application	Dilution
1	AKT	Cell signaling	#9272	Rabbit	P	60	WB	1:1000
2	phospho-AKT (Ser-473)	Cell signaling	#4058	Rabbit	M	60	WB	1:1000
3	AMPKα	Cell signaling	#5831	Rabbit	M	62	WB	1:1000
4	phospho-AMPKα (Thr-172)	Cell signaling	#2535	Rabbit	M	62	WB	1:1000
5	Atg5	Cell signaling	#12994	Rabbit	M	55	WB	1:1000
6	β-actin	Sta Cruz Biotechnology	sc-81178	Mouse	M	45	WB	1:10000
7	β-actin (HRP Conjugate)	Cell signaling	#12620	Rabbit	M	45	WB	1:1000
8	Beclin-1	Cell signaling	#4122	Mouse	M	60	WB	1:1000
9	Caspase-3	Cell signaling	#9662	Rabbit	P	17, 19, 35	WB	1:1000
10	Cleaved Caspase-8 (Asp-391)	Cell signaling	#9496	Rabbit	M	18, 41, 43	WB	1:1000
11	Ki67	Abcam	ab15580	Rabbit	P	-	IHC	1:100
12	LC3B	Cell signaling	#2775	Rabbit	P	14, 16	WB, IF	1:1000, 1:100
13	phospho-p44/42 MAPK (Erk1/2) (Thr-202/Tyr-204)	Cell signaling	#4376	Rabbit	M	42, 44	WB	1:1000
14	p44/42 MAPK (Erk1/2)	Cell signaling	#4695	Rabbit	M	42, 44	WB	1:1000
15	phospho-p44/42 MAPK (Erk1/2) (Thr-202/Tyr-204) Biotinylated	Cell signaling	#4094	Rabbit	M	42,44	WB	1:1000
16	p44/42 MAPK (Erk1/2) Biotinylated	Cell signaling	#5013	Rabbit	M	42,44	WB	1:1000
17	MEK1/2	Cell signaling	#8727	Rabbit	M	45	WB	1:1000
18	phospho-MEK1/2 (Ser-217/Ser-221) Biotinylated	Cell signaling	#8727	Rabbit	M	45	WB	1:1000
19	phospho-mTOR (Ser-2448)	Cell signaling	#2971	Rabbit	P	289	WB	1:1000
20	mTOR	Cell signaling	#2972	Rabbit	P	289	WB	1:1000
21	NTAL	Cell signaling	#9533	Rabbit	P	25	WB,	1:1000
22	NTAL Biotinylated	R&D Systems	BAF4066	Sheep	P	25	WB	1:250
23	NTAL	Sigma	HPA003462	Rabbit	P	26.6	IHC	1:50
24	PARP	Cell signaling	#9542	Rabbit	P	89, 116	WB	1:1000
25	PI3 Kinase Class III	Cell signaling	#4263	Rabbit	M	100	WB	1:1000
26	Ras	Cell signaling	#3339	Rabbit	M	21	WB	1:1000
27	Ras	Cell signaling	#8832	Mouse	M	21	WB	1:200
28	Raptor	Cell signaling	#2280	Rabbit	M	150	WB	1:1000
29	SQSTM1/p62	Cell signaling	#5114	Rabbit	P	62	WB	1:1000
30	β-tubulin	Cell signaling	#2146	Rabbit	P	55	WB	1:1000

* P = Polyclonal; M = Monoclonal; WB = Western Blotting, IHC= Immunohistochemistry, IF = Immunofluorescence

Supplementary Results

NTAL Knockdown promotes lysosomal accumulation and autophagic flux

Lysosomes are the key organelles for autophagic degradation [1]. We examined whether NTAL-KD affects lysosome accumulation by analyzing abundance of acidic vesicular organelles. The NB4 (CT and NTAL-KD) cells were treated with chloroquine (Clq), which inhibits autophagosome-lysosome fusion, blocking the degradation of LC3-II and leading to its accumulation [2]; rapamycin (Rapa), which induces autophagy through the mTOR-dependent signaling pathway [3]; bafilomycin A1 (BafA1), which prevents maturation of autophagic vacuoles by inhibiting fusion between autophagosomes and lysosomes [4], or is kept in FBS-free medium to induce autophagy through starvation. NTAL–KD NB4 cells presented an increased lysosomal accumulation. Treatment with Clq, Rapa, or serum starvation potentiated NTAL-KD lysosomal accumulation and treatment with BafA1 decreased lysosome accumulation in NB4 cells (Supplementary Figure S2B). To test if NTAL modulates the autophagic flux, NTAL-KD and treatments with Clq, Rapa or serum starvation were evaluated by western blotting. The Atg5 autophagy marker levels increased with NTAL-KD, and further increased after all the treatments. Levels of the LC3-II, protein presented a reduction in NB4 cells after the treatments, supporting an increased autophagic flux (Supplementary Figure S2C). These results were supported by immunofluorescent analysis of total LC3 levels (Supplementary Figure S2D).

Effect of autophagy inhibition in NTAL-KD NB4 cells

To determine the effect of autophagy inhibition, NB4 (CT and NTAL-KD) cells were treated with 3-methyladenine (3-MA), with an effect on class III PI3K activity[5]. Treatment with 3-MA reduced the autophagic flux over a period of 6 h as indicated by

the accumulation of LC3-I, suggesting the blocking of LC3 lipidation. This effect was sustained even 18 h after the removal of 3-MA (Supplementary Figure S3A).

NTAL knockdown leads to mitochondrial alterations

We used transmission electron microscopy to assess morphological alterations at ultrastructural level promoted by NTAL-KD. NB4 NTAL-KD cells presented a decrease in cytoplasmic vacuolization with the presence of mitochondrial degeneration (Supplementary Figure S3B).

Characterization of the NB4 cell graft in NSG mice

No difference was observed between the histological characteristics of engrafted tumors formed by control and NTAL-KD cells (Supplementary Figure S4A). NTAL-KD was confirmed by immunostaining for NTAL (Supplementary Figure S4B).

Supplementary Information: Figures Legends

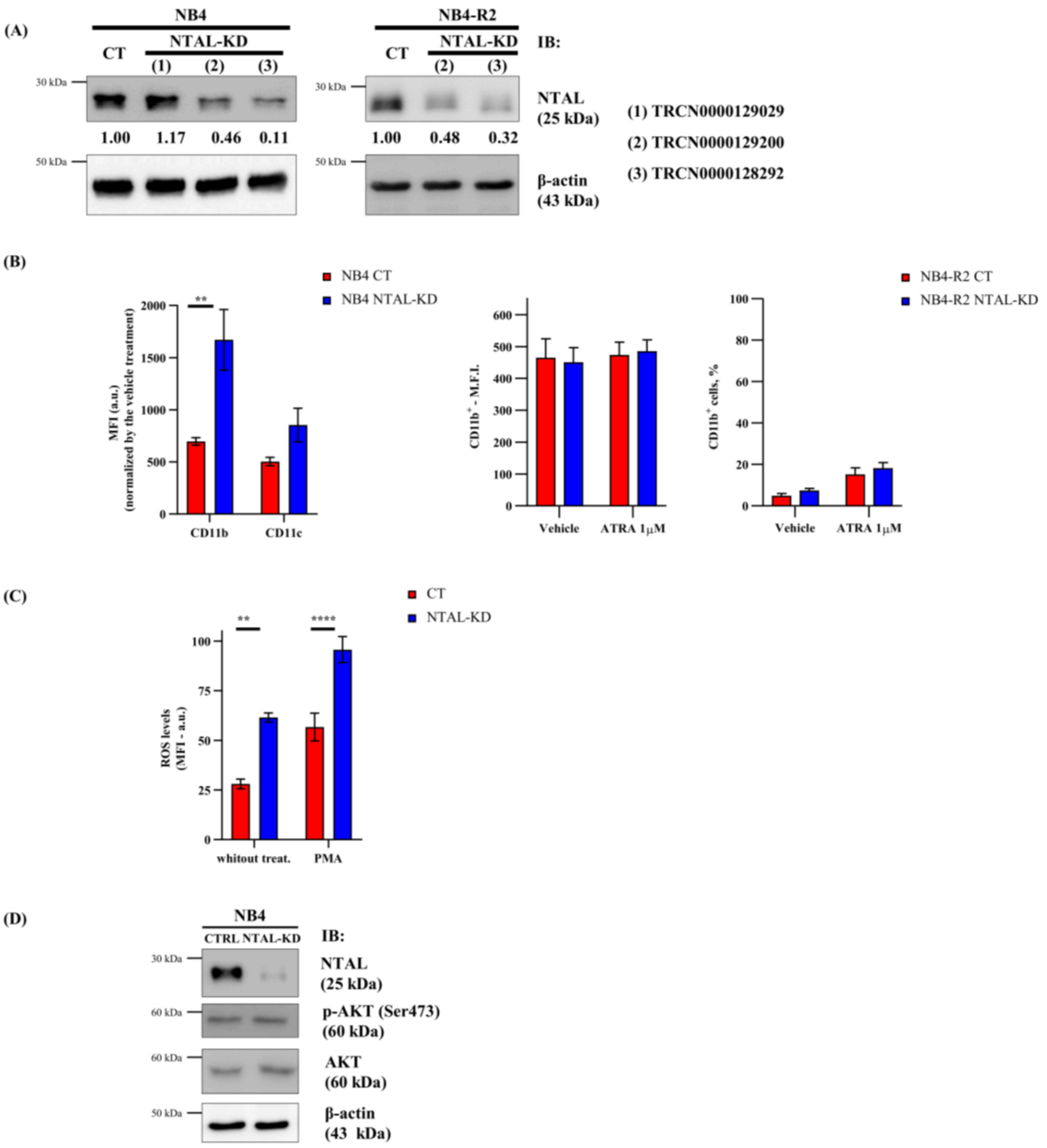

Supplementary Figure S1: (A) NTAL knockdown was obtained from NB4 and NB4-R2 cells via lentiviral transduction of shRNA that targets NTAL. Samples 1, 2 and 3 are cell lines generated with different shRNA sequences. The NB4 cell line identified as (3) was used for further study because of its greater extent of knockdown. Bar graphs present the Mean Fluorescence Intensity for cell lines transduced evaluated by flow cytometry **(B)** of CD11b and CD11c expression in NB4 and NB4 R2 cells (CT [control] and NTAL-KD) after 72 h of ATRA (1 µM) stimulation for differentiation and **(C)**

General ROS accumulation using (2’,7’-dichlorofluorescein diacetate [H_2DCFDA] fluorescence) in NB4R2 cells (CT [control] and NTAL-KD). **(D)** Effect of knockdown of the NTAL protein in NB4 cells (CT or NTAL-KD) on the AKT pathway.

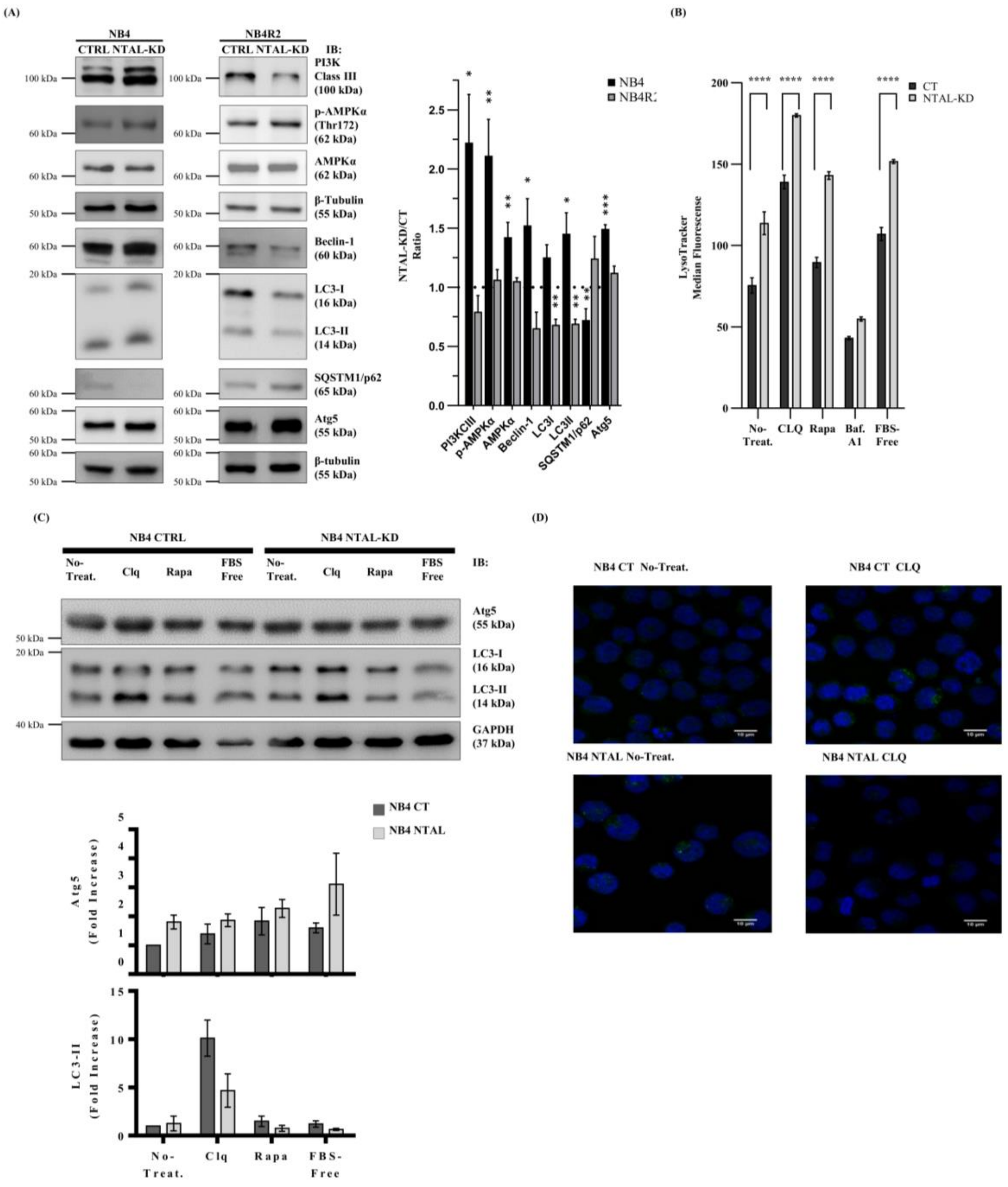

Supplementary Figure S2: NTAL knockdown modulates markers of autophagy. **(A)** Effect of knockdown of the NTAL protein in NB4 and NB4R2 (CT or NTAL-KD) on the components of autophagy: PI3K class III, p-AMPKα (Thr-172), AMPKα, Beclin-1,

LC3-I, LC3-II, SQSTM1/p62 and ATG5. **(B)** Formation of acidic vesicular organelles in the NB4 (CT and NTAL-KD) cells evaluated by LysoTracker Green DND-26 staining under different conditions (10 µM, chloroquine (Clq); 1 µM, rapamycin (Rapa); 10 nM, bafilomycin A1 (BafA1) and starvation by FBS-free medium) for 16-18 h measured by flow cytometry. Values are shown as the median ±SD (**** P<0.0001). **(C)** Semi-quantitative analysis of the effect of treatment with 10 µM Clq or 1 µM Rapa or starvation by FBS-free medium for 16-18 h on the alteration of autophagic flux markers in NB4 (CT and NTAL-KD) cells evaluated by western blotting. **(D)** Accumulation of LC3 puncta in NB4 (CT and NTAL-KD) cells with 30 µM chloroquine (Clq) for 16-18 h. The distribution of LC3 was examined by immunofluorescence.

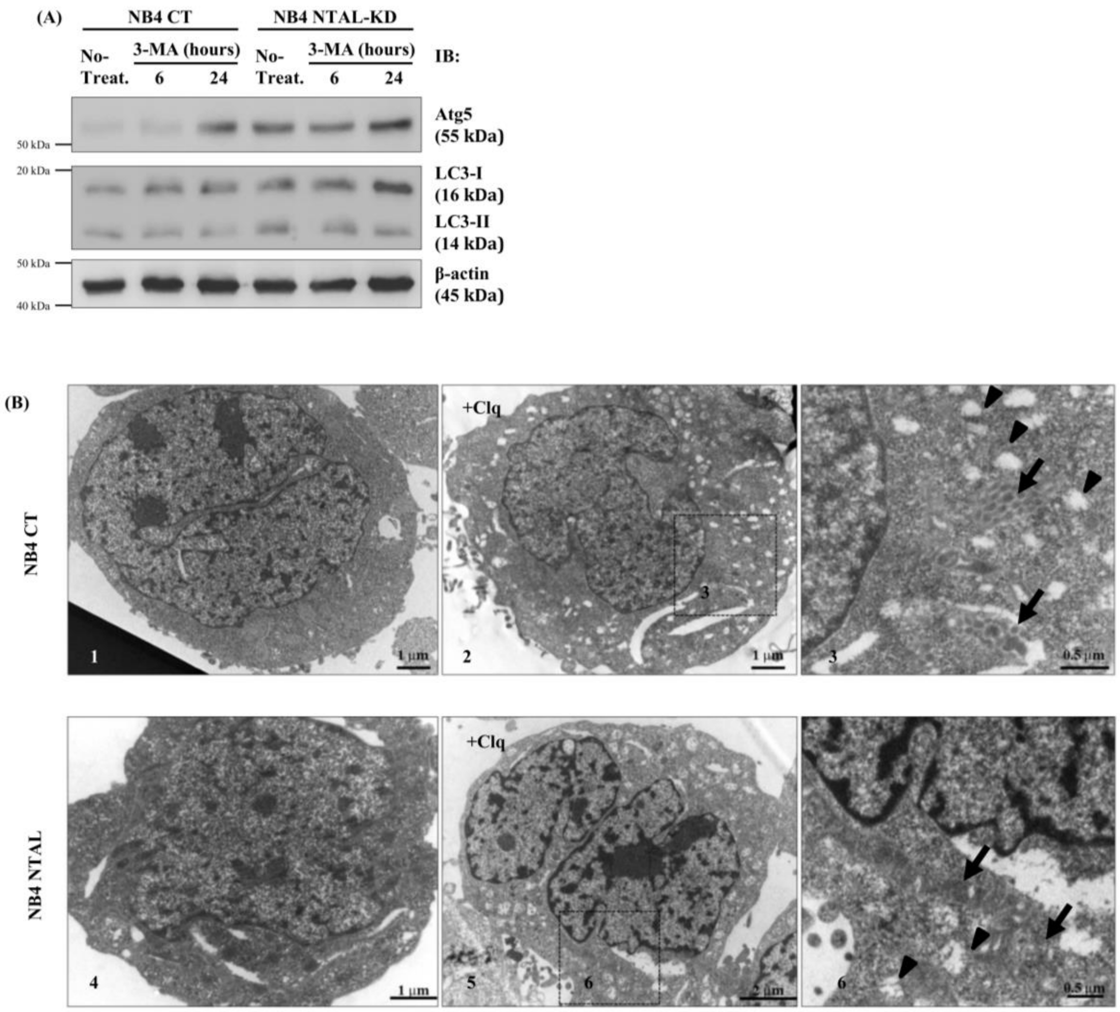

Supplementary Figure S3: (A) Analysis of autophagy markers in NB4 cells exposed to 3-methyladenine (3-MA) (5 mM) for 6 h. Cells were washed for evaluation of 3-MA effects for extra 18 h. **(B)** NTAL knockdown affects cellular structures and mitochondrial function. Transmission electron microscopy was performed in the NB4 (CT and NTAL-KD) cells treated or not- with 10 µM chloroquine (Clq) for 16-18 h to examine cell structures consistent with cellular stress, such as vesicles (◀) and potential mitophagy (←).

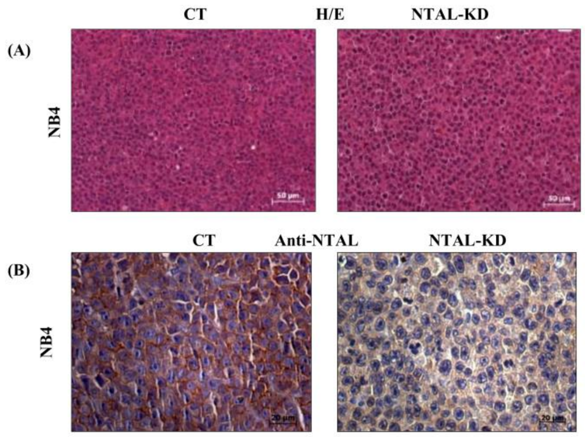

Supplementary Figure S4: Female 12-week-old NSG mice were injected subcutaneously into the left thigh with 1 x10^6 NTAL-KD cells and received an equal number of CT cells in the right thigh subcutaneously. The cells in the right and left thigh show tumors stained sections with **(A)** hematoxylin and eosin, or **(B)** immunostained for NTAL.

Supplementary References

1 Shen, H. M. & Mizushima, N. At the end of the autophagic road: an emerging understanding of lysosomal functions in autophagy. *Trends Biochem Sci* **39**, 61-71, doi:10.1016/j.tibs.2013.12.001 (2014).

2 Harhaji-Trajkovic, L. *et al.* Chloroquine-mediated lysosomal dysfunction enhances the anticancer effect of nutrient deprivation. *Pharm Res* **29**, 2249-2263, doi:10.1007/s11095-012-0753-1 (2012).

3 Tanemura, M. *et al.* Rapamycin causes upregulation of autophagy and impairs islets function both in vitro and in vivo. *Am J Transplant* **12**, 102-114, doi:10.1111/j.1600-6143.2011.03771.x (2012).

4 Yamamoto, A. *et al.* Bafilomycin A1 prevents maturation of autophagic vacuoles by inhibiting fusion between autophagosomes and lysosomes in rat hepatoma cell line, H-4-II-E cells. *Cell Struct Funct* **23**, 33-42 (1998).

5 Wu, Y. T. *et al.* Dual role of 3-methyladenine in modulation of autophagy via different temporal patterns of inhibition on class I and III phosphoinositide 3-kinase. *J Biol Chem* **285**, 10850-10861, doi:10.1074/jbc.M109.080796 (2010).

Full images of membranes of western blottings

Figure 1A – Upper panel – NTAL (25 kDa)

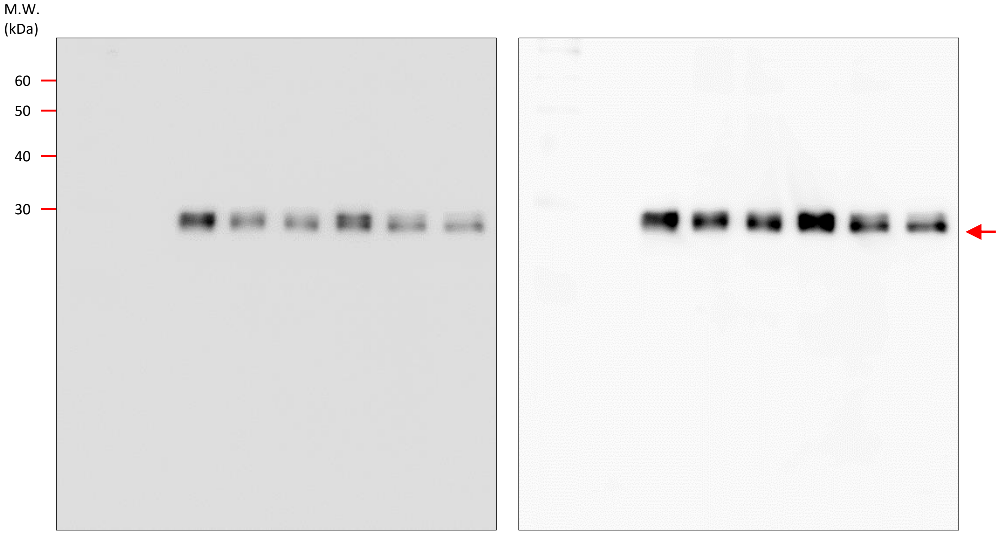

Figure 1A – Upper panel – β-tubulin (55 kDa)

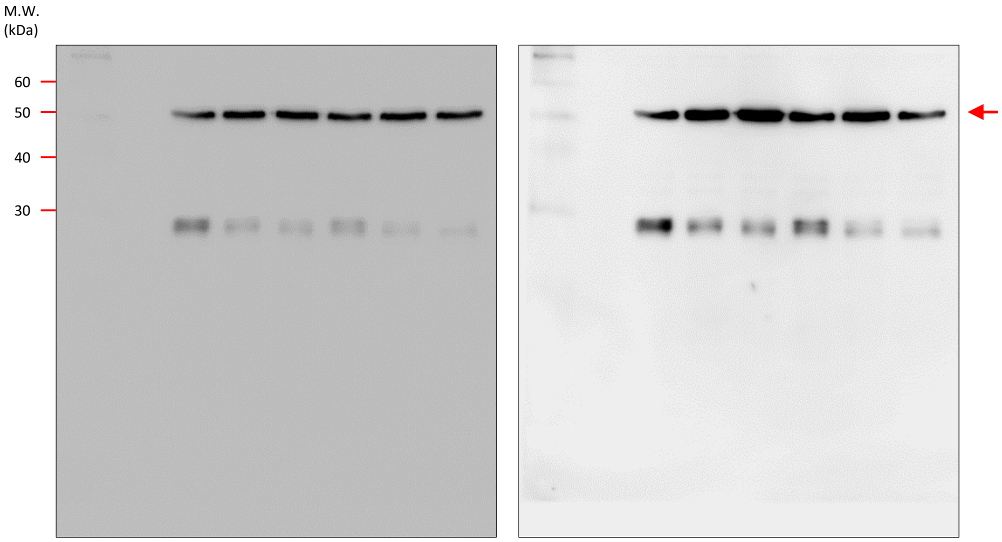

Figure 1A – Lower panel – NTAL (25 kDa)

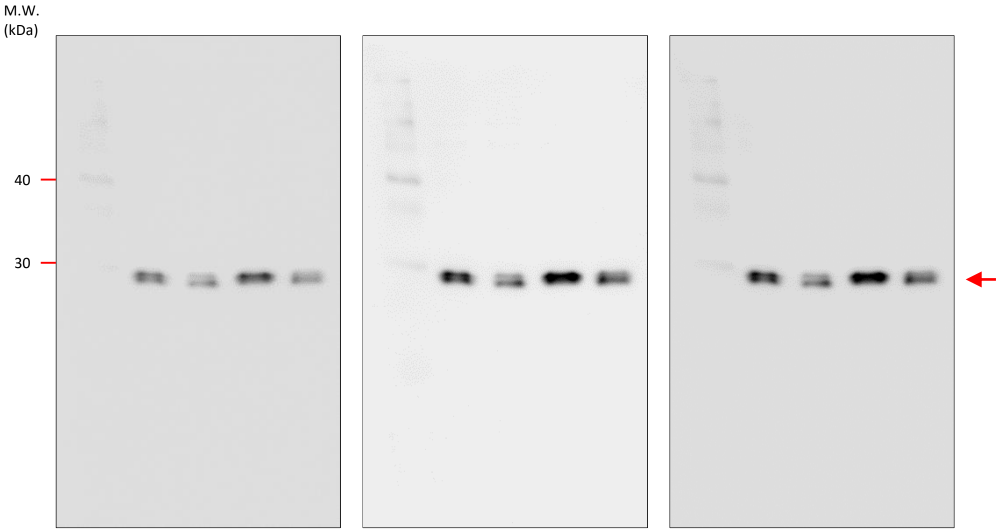

Figure 1A – Lower panel – β-tubulin (55 kDa)

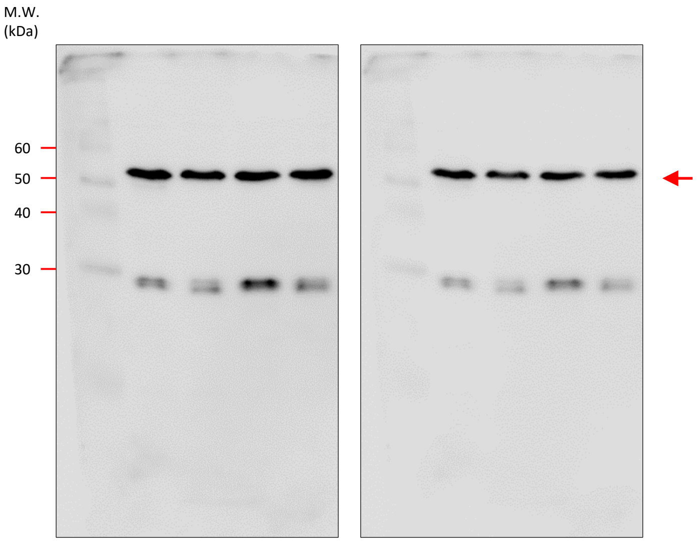

Figure 1C – NB4 – NTAL (25 kDa)

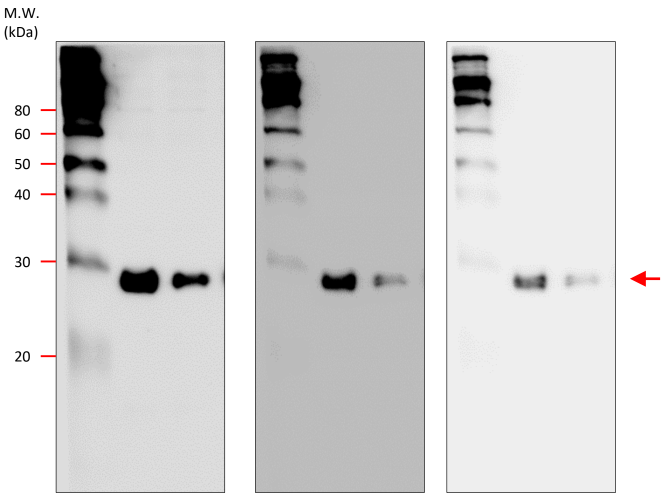

Figure 1C – NB4 –Cleaved Caspase-8 (43 and 41kDa)

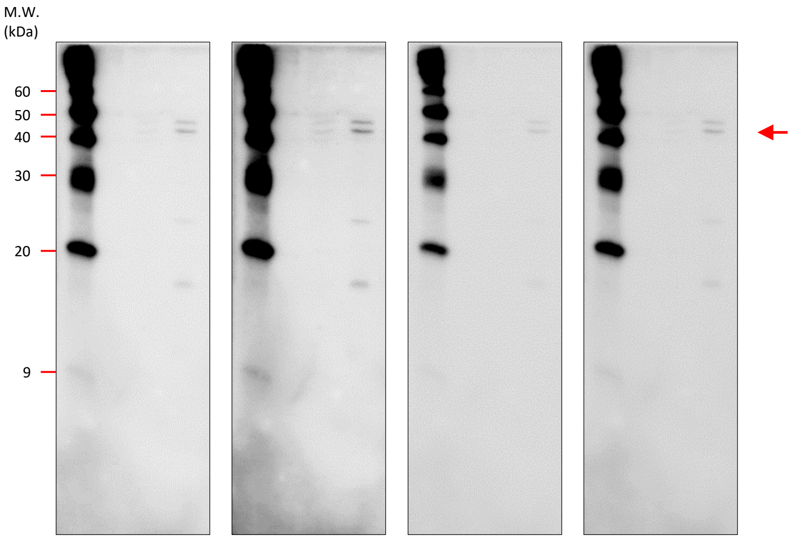

Figure 1C – NB4 – Caspase-3 (35 kDa)

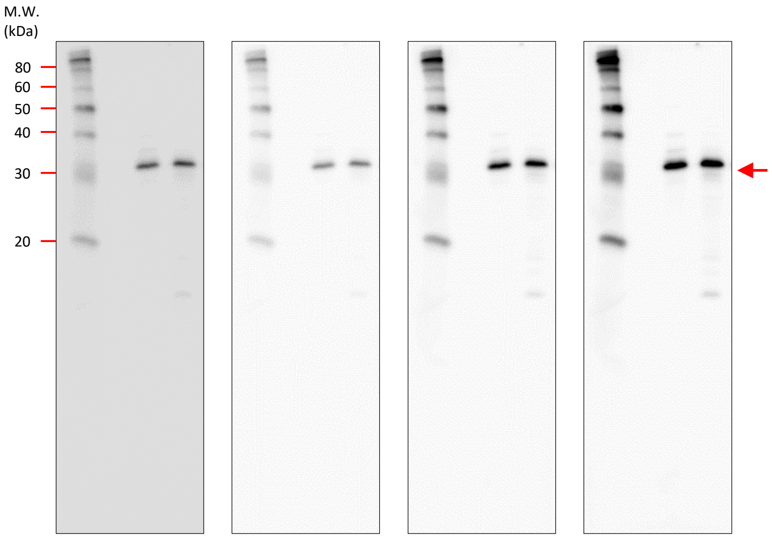

Figure 1C – NB4 – β-tubulin (55 kDa)

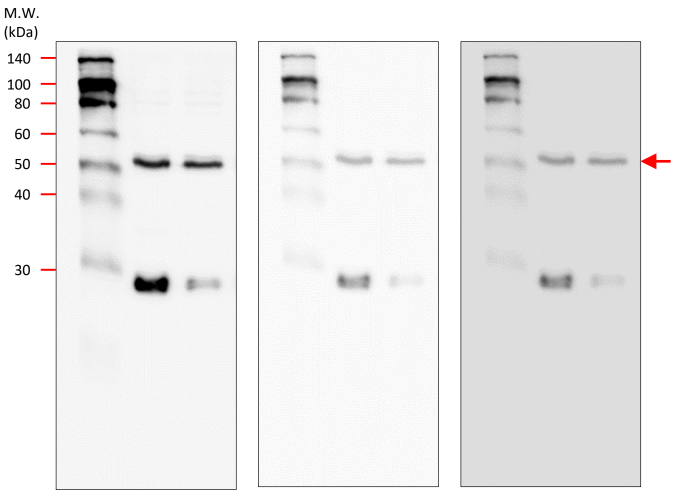

Figure 1C – NB4 R2 – NTAL (25 kDa)

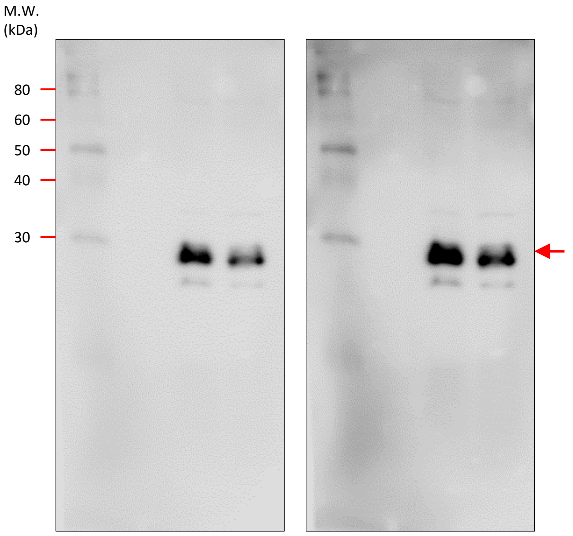

Figure 1C – NB4 R2 – Cleaved Caspase-8 (43 and 41kDa)

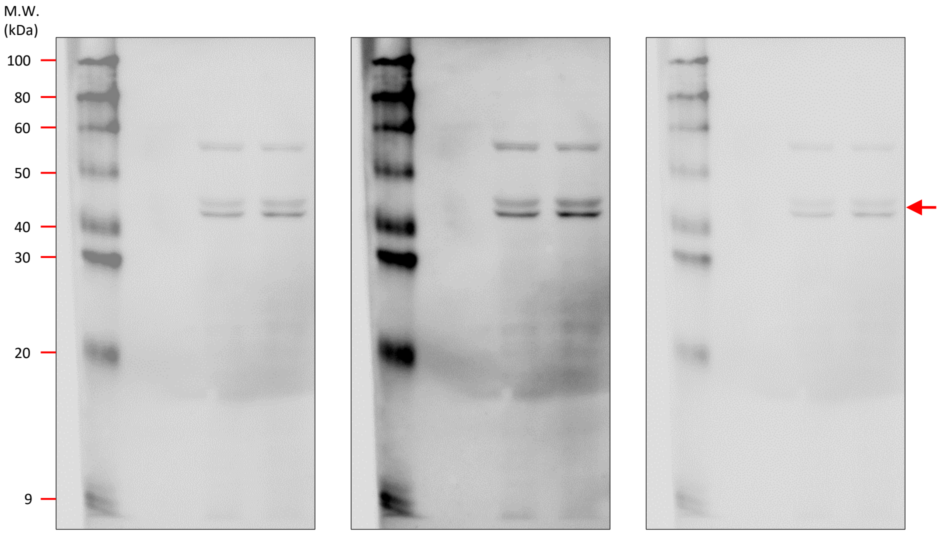

Figure 1C – NB4 R2 – Caspase-3 (35 kDa)

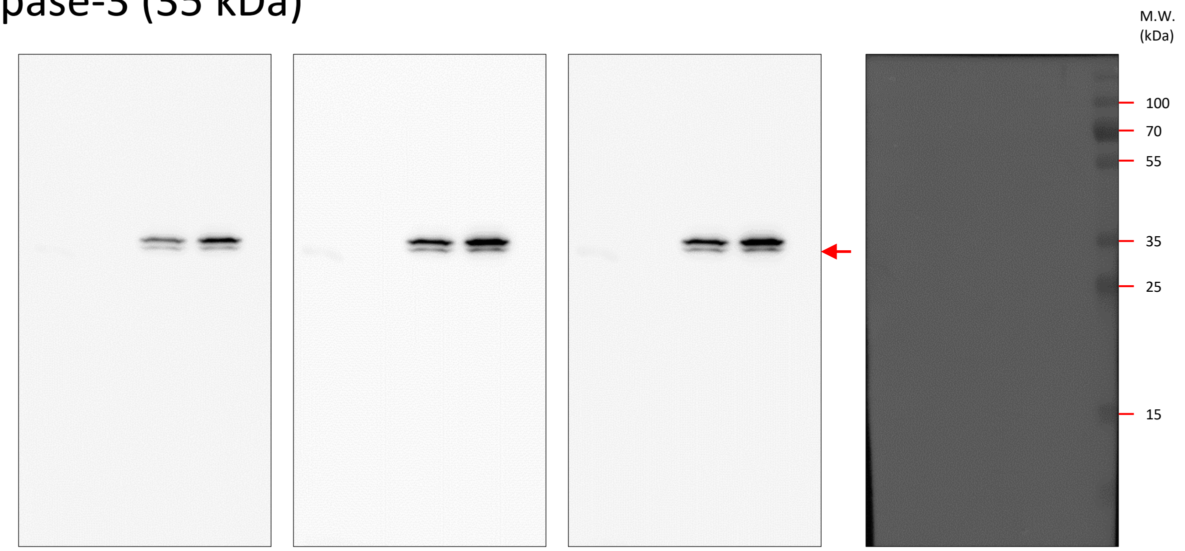

Figure 1C – NB4 R2 – β-tubulin (55 kDa)

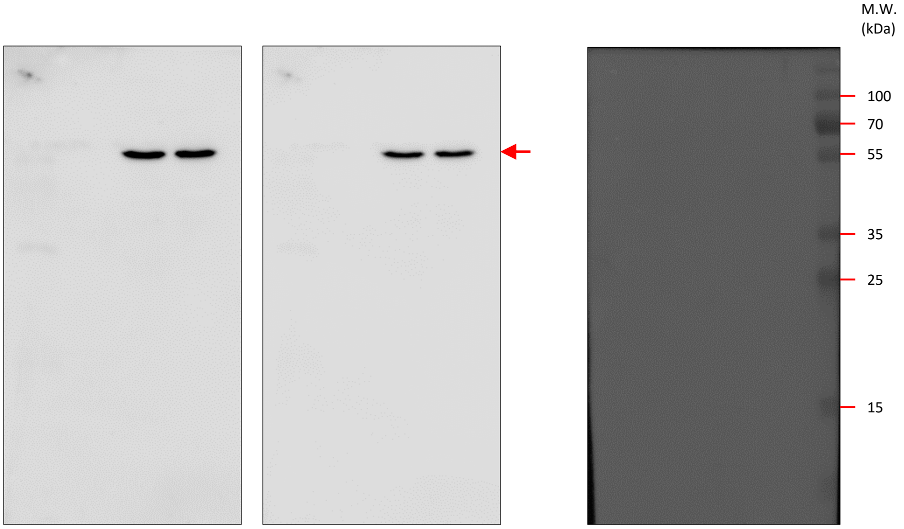

Figure 1D – NB4 – PARP and Cleaved PARP (116, 89 kDa)

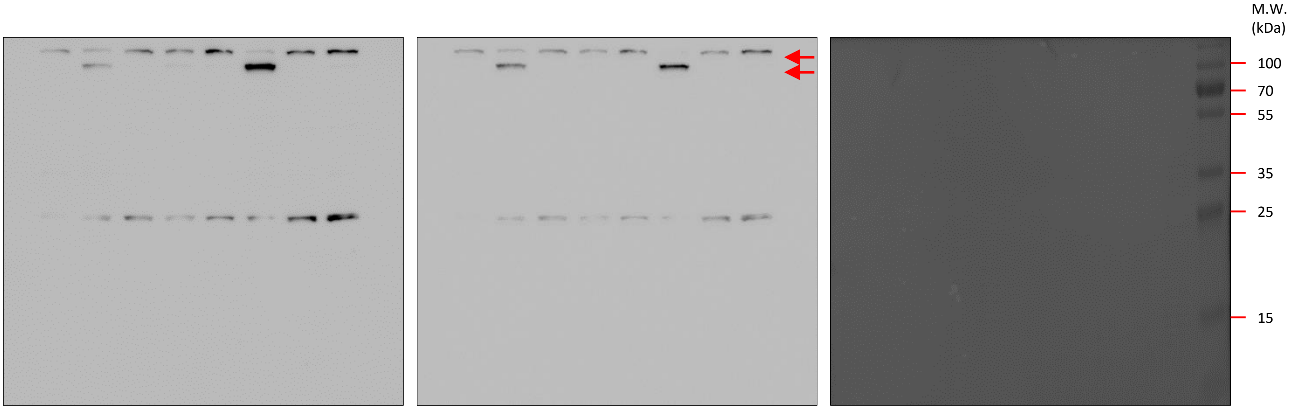

Figure 1D – NB4 – Cleaved Caspase-8 (43 and 41 kDa)

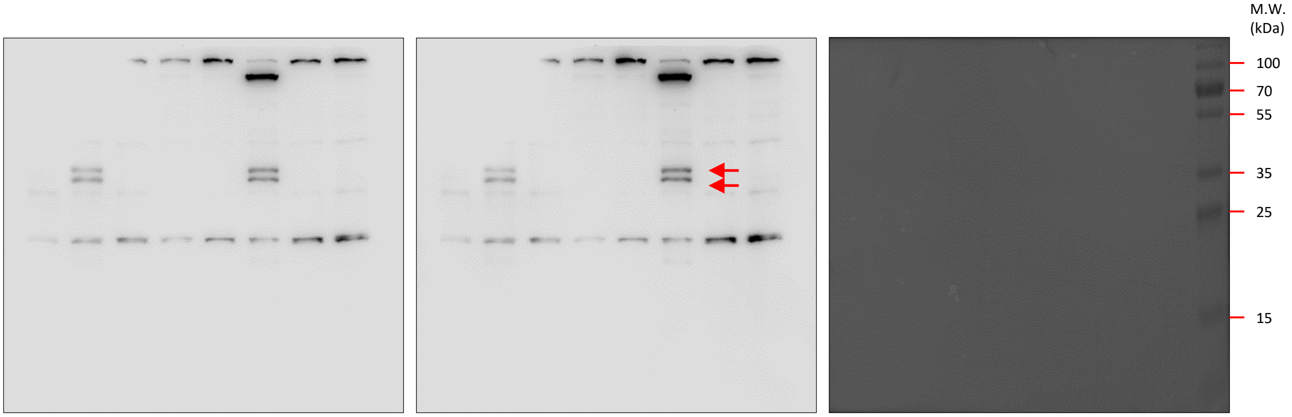

Figure 1D – NB4 – β-actin (45 kDa)

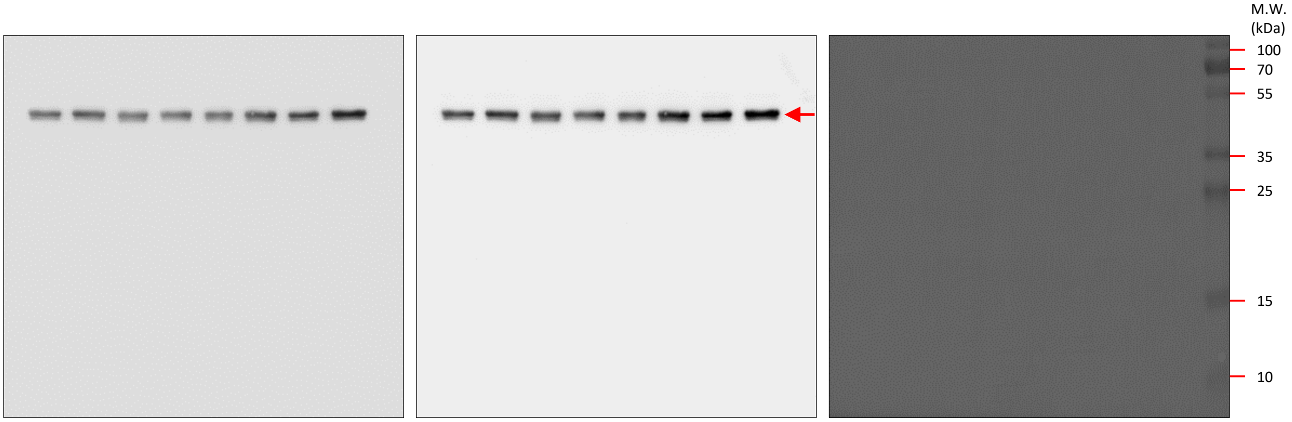

Figure 2A – NB4 – NTAL (25 kDa)

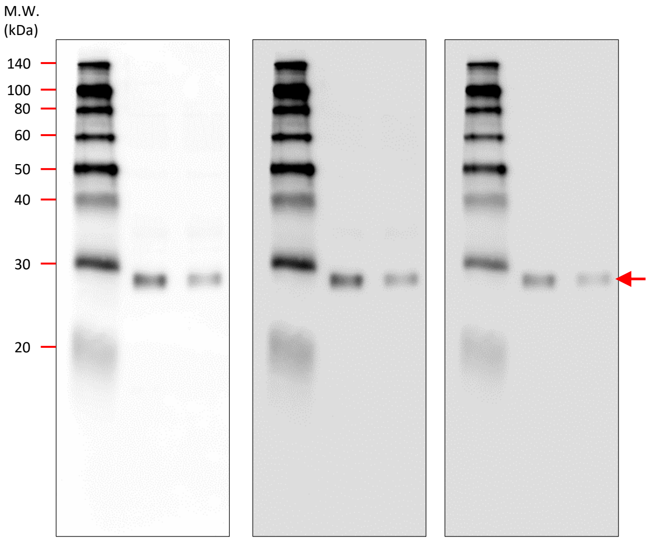

Figure 2A – NB4 – Raptor (150 kDa)

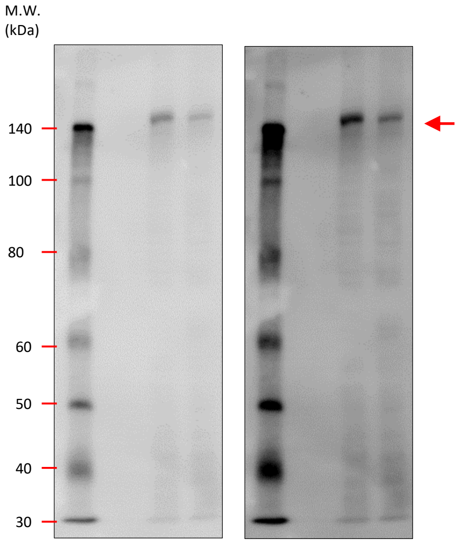

Figure 2A – NB4 – p-mTOR (S-2481)(289 kDa)

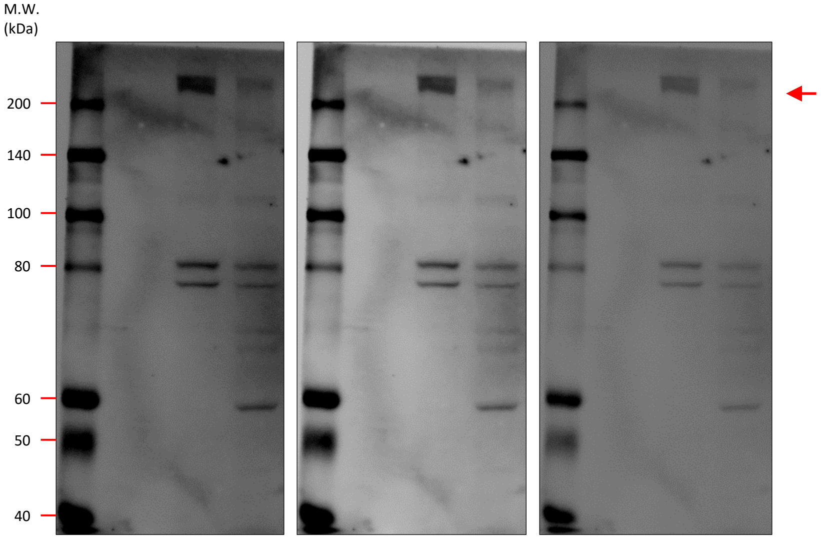

Figure 2A – NB4 – mTOR (289 kDa)

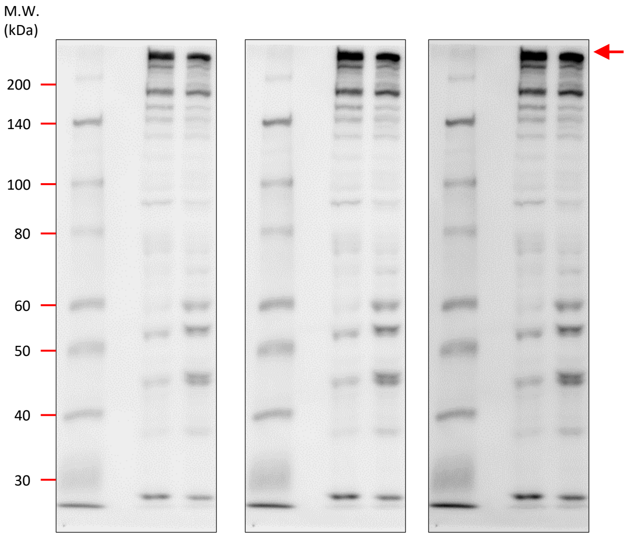

Figure 2A – NB4 – β-actin (45 kDa)

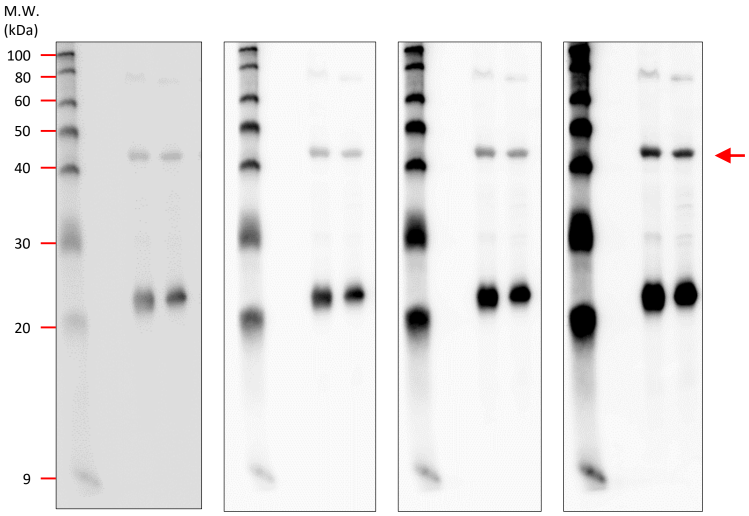

Figure 2A – NB4-R2 – NTAL (25 kDa)

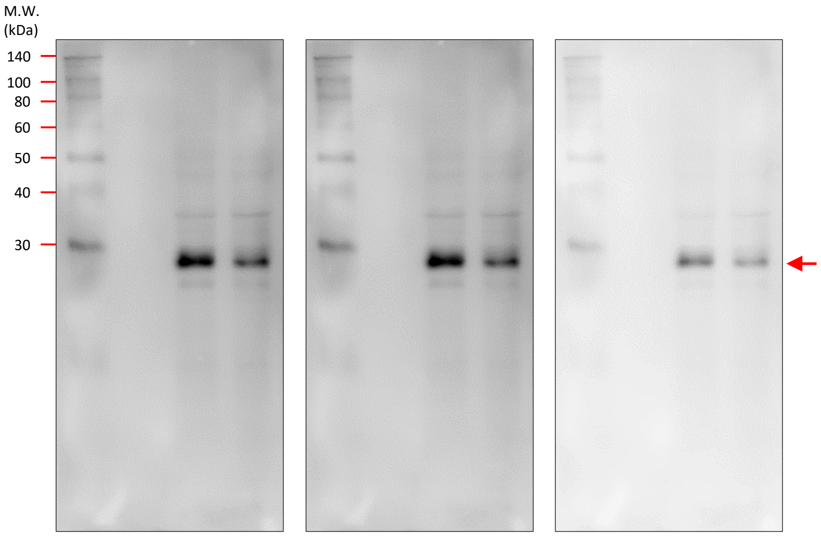

Figure 2A – NB4-R2 – Raptor (150 kDa)

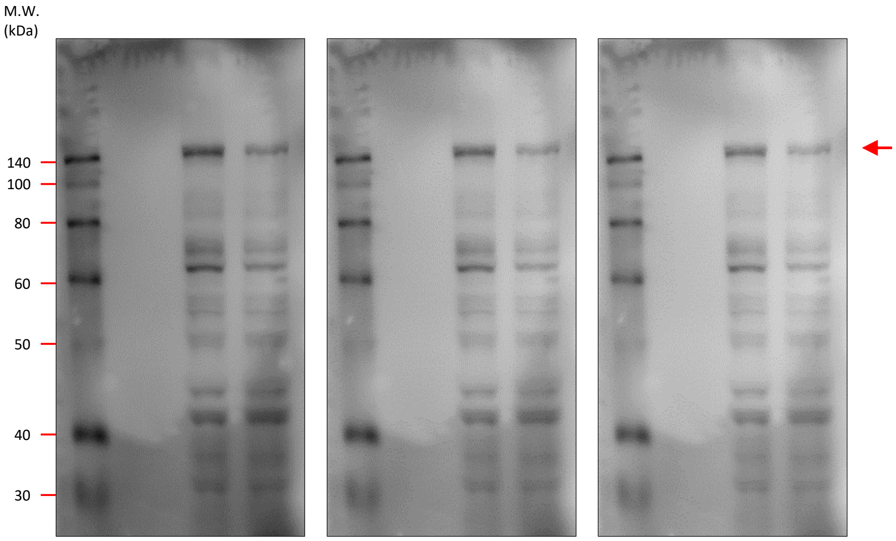

Figure 2A – NB4-R2 – p-mTOR (S-2481)(289 kDa)

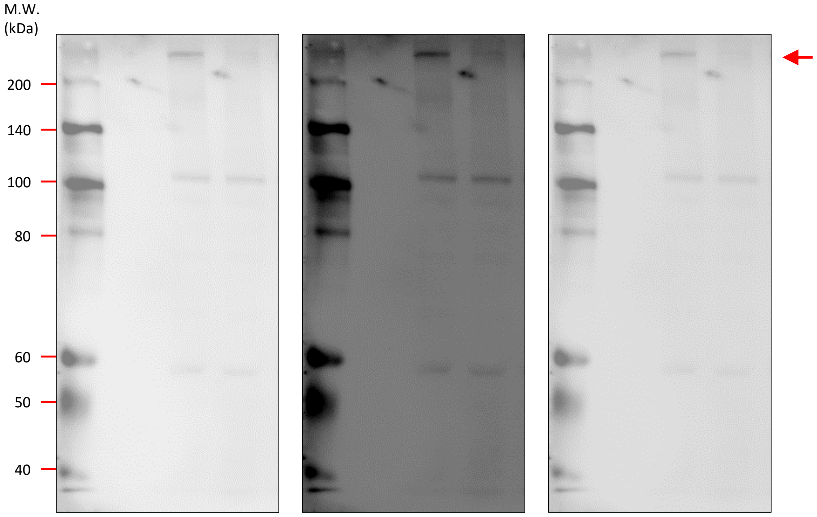

Figure 2A – NB4-R2 – mTOR (289 kDa)

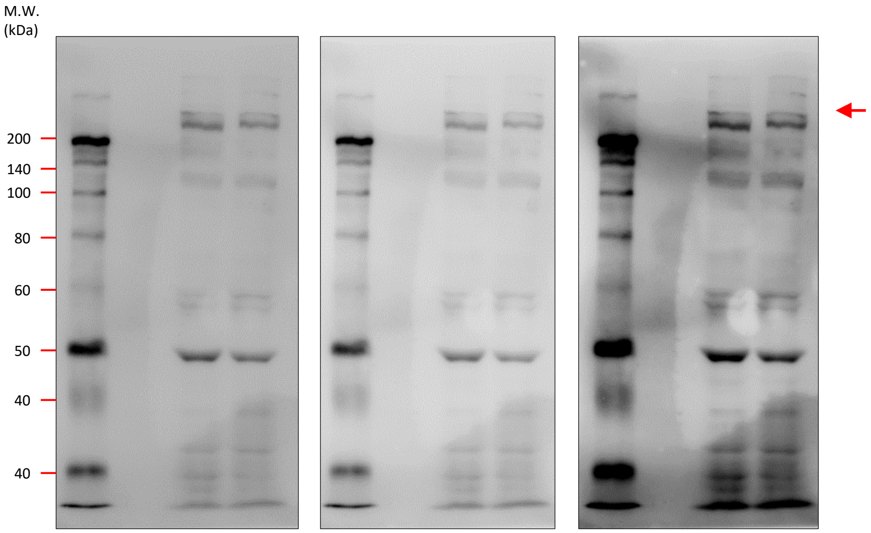

Figure 2A – NB4-R2 – β–actin (45 kDa)

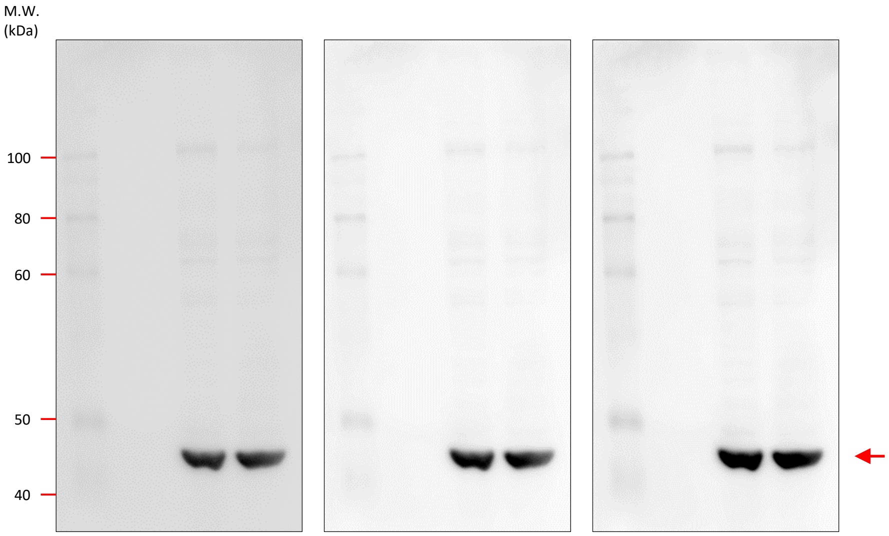

Figure 2C – NB4 – Ras (21 kDa)

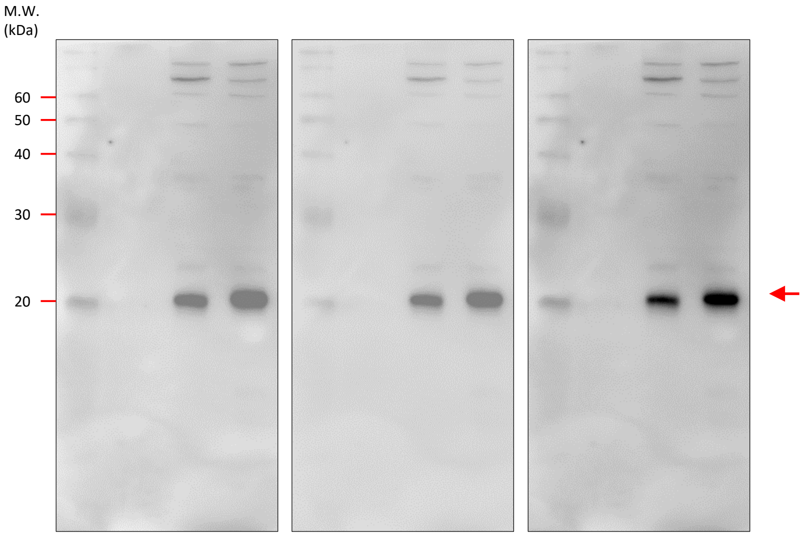

Figure 2C – NB4 – p-MEK1/2 (S-217/221)(45 kDa)

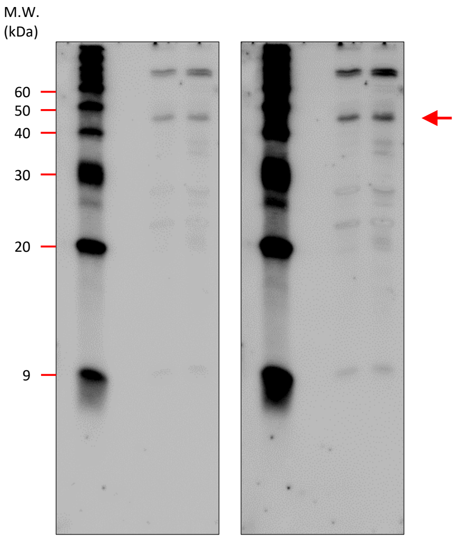

Figure 2C – NB4 – MEK1/2 (45 kDa)

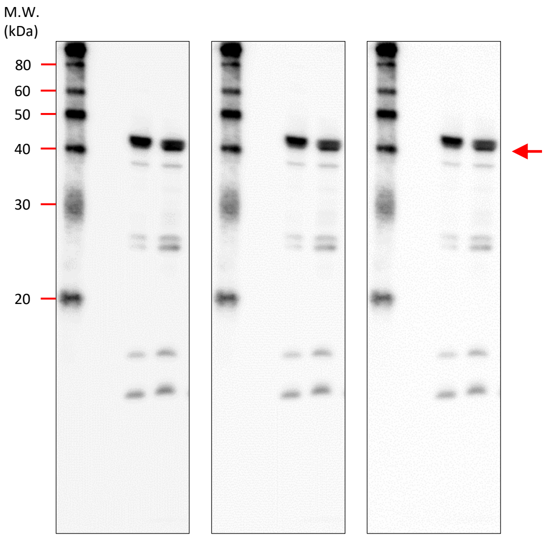

Figure 2C – NB4– p-p44/42 MAPK (Erk1/2)(T-202/Y-204) (44, 42 kDa)

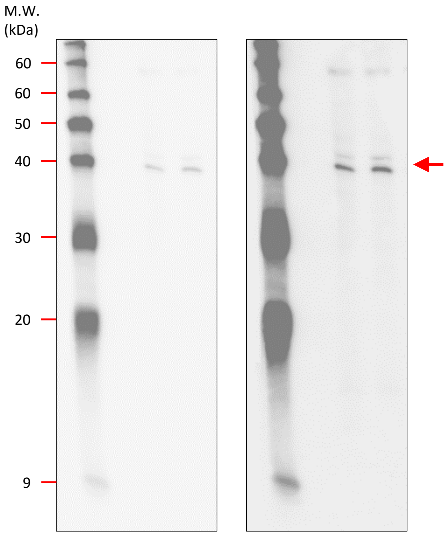

Figure 2C – NB4 – p44/42 MAPK (Erk1/2) (44, 42 kDa)

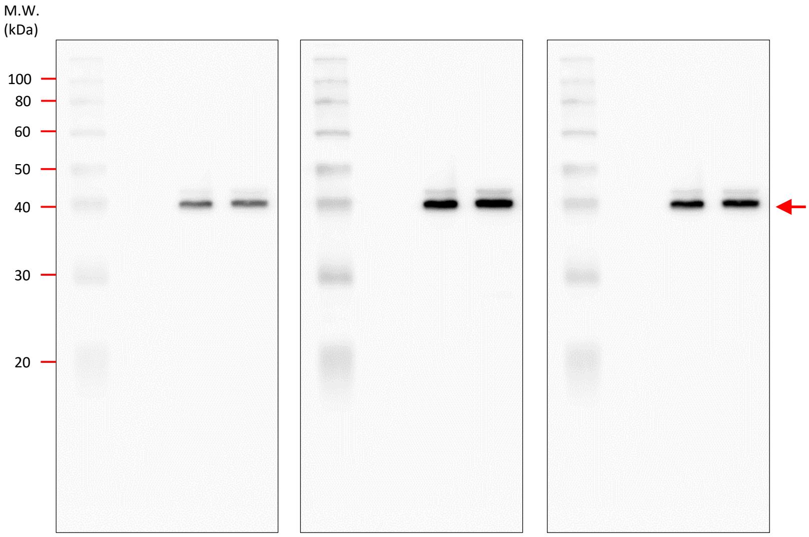

Figure 2C – NB4 – β-actin (45 kDa)

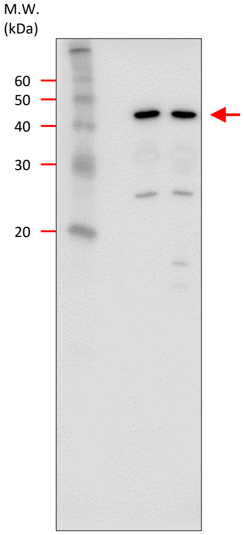

Figure 2C – NB4-R2 – Ras (21 kDa)

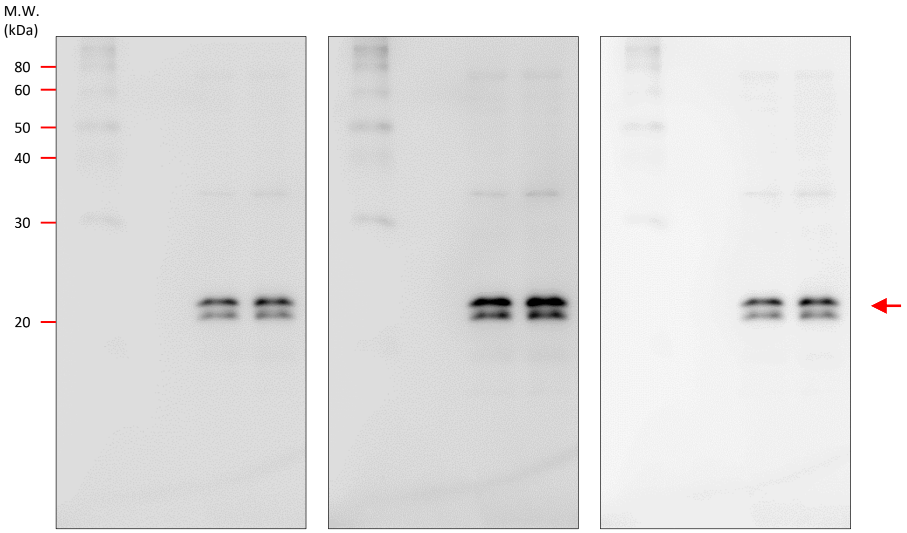

Figure 2C – NB4-R2 – p-MEK1/2 (S-217/221)(45 kDa)

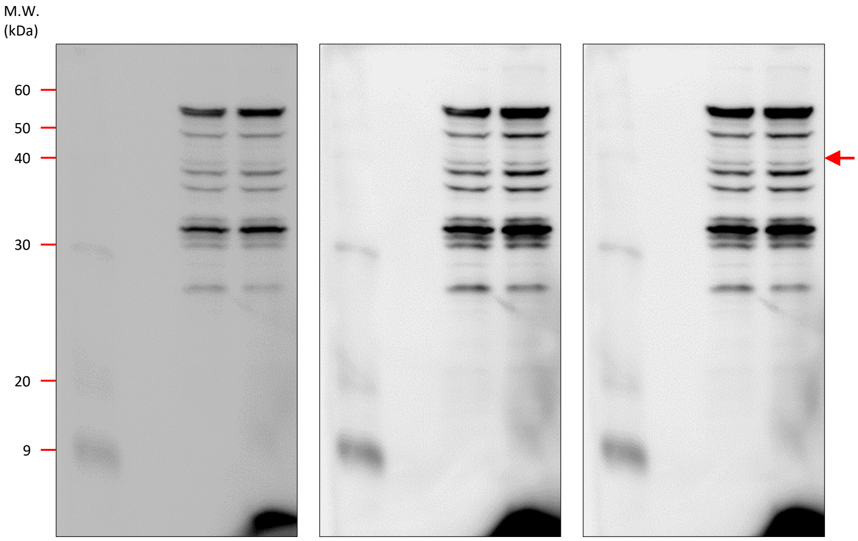

Figure 2C – NB4-R2 – MEK1/2(45 kDa)

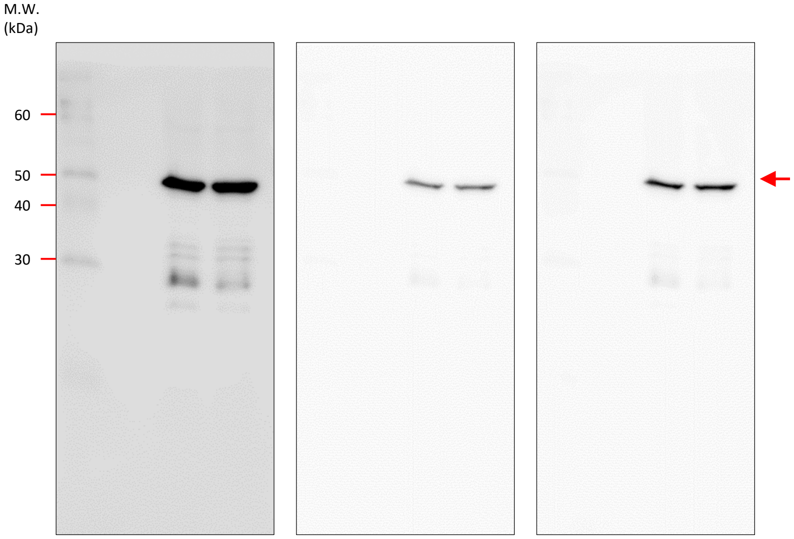

Figure 2C – NB4-R2 – p-p44/42 MAPK(Erk1/2) (T-202/Y-204)(44, 42 kDa)

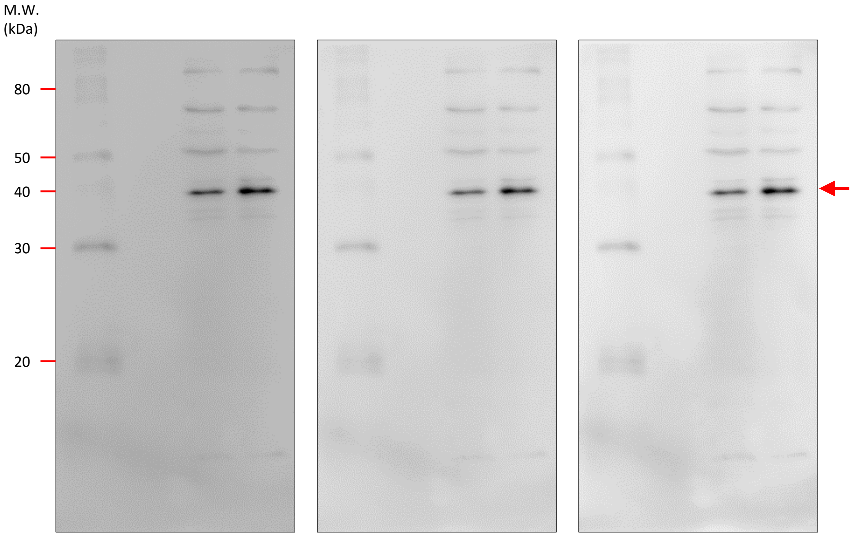

Figure 2C – NB4-R2 – p44/42 MAPK(Erk1/2) (44, 42 kDa)

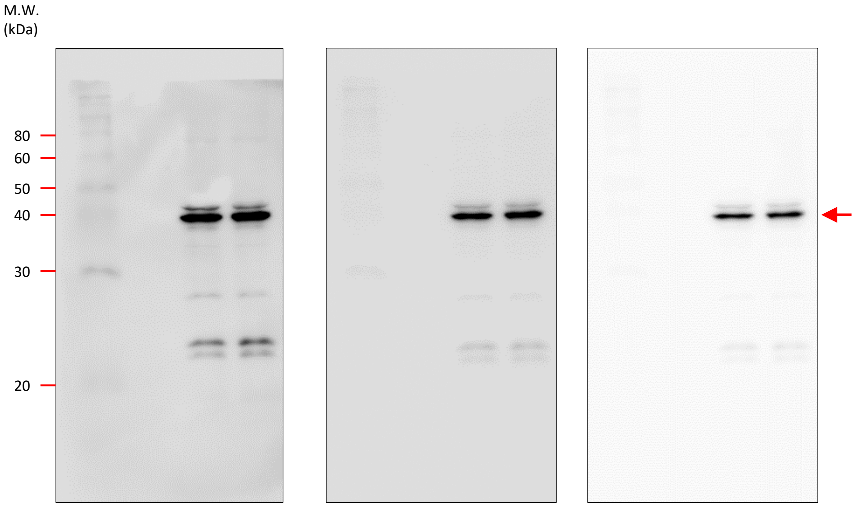

Figure 2C – NB4-R2 – β-actin (45 kDa)

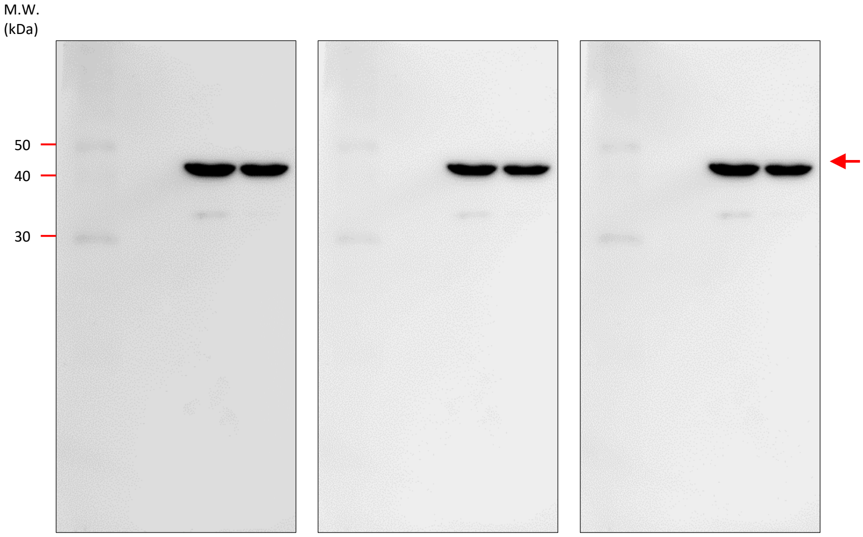

Figure 3E – NB4 XENOGRAFT MOUSE MODEL –
NTAL (25 kDa)

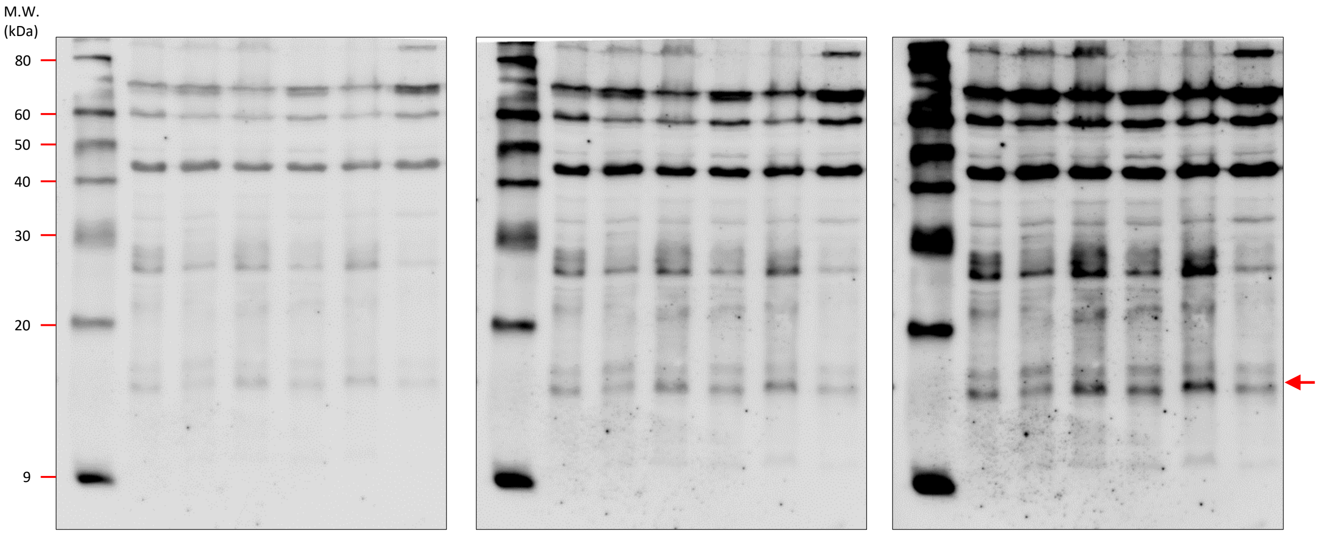

Figure 3E – NB4 XENOGRAFT MOUSE MODEL –
Ras (21 kDa)

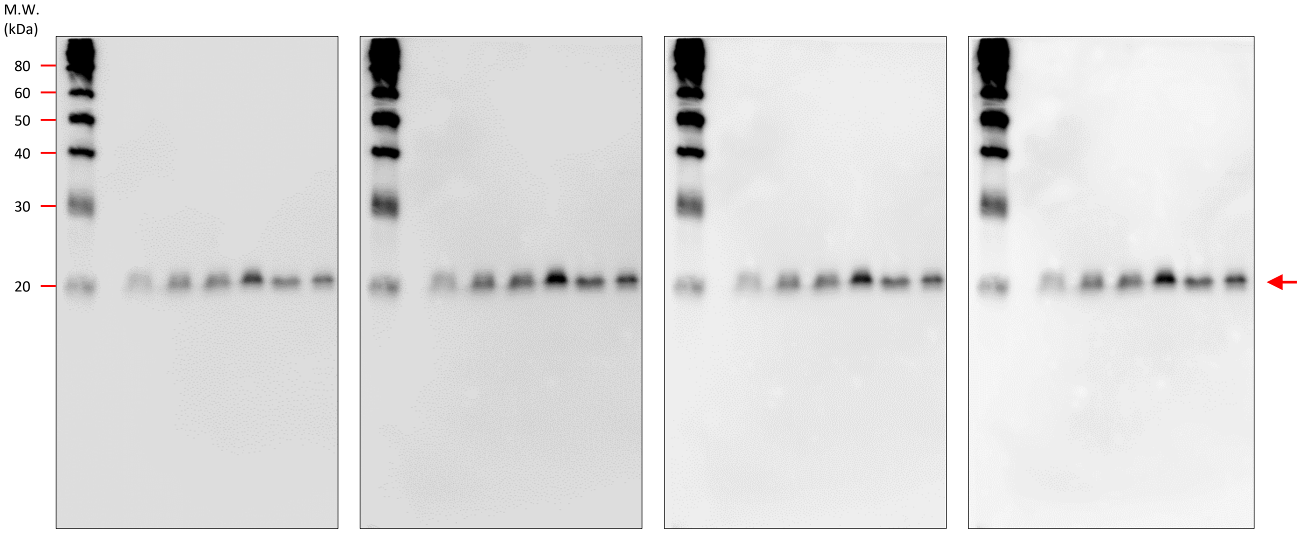

Figure 3E – NB4 XENOGRAFT MOUSE MODEL –
p-p44/42 MAPK(Erk1/2)(T202/Y204) (44, 42 kDa)

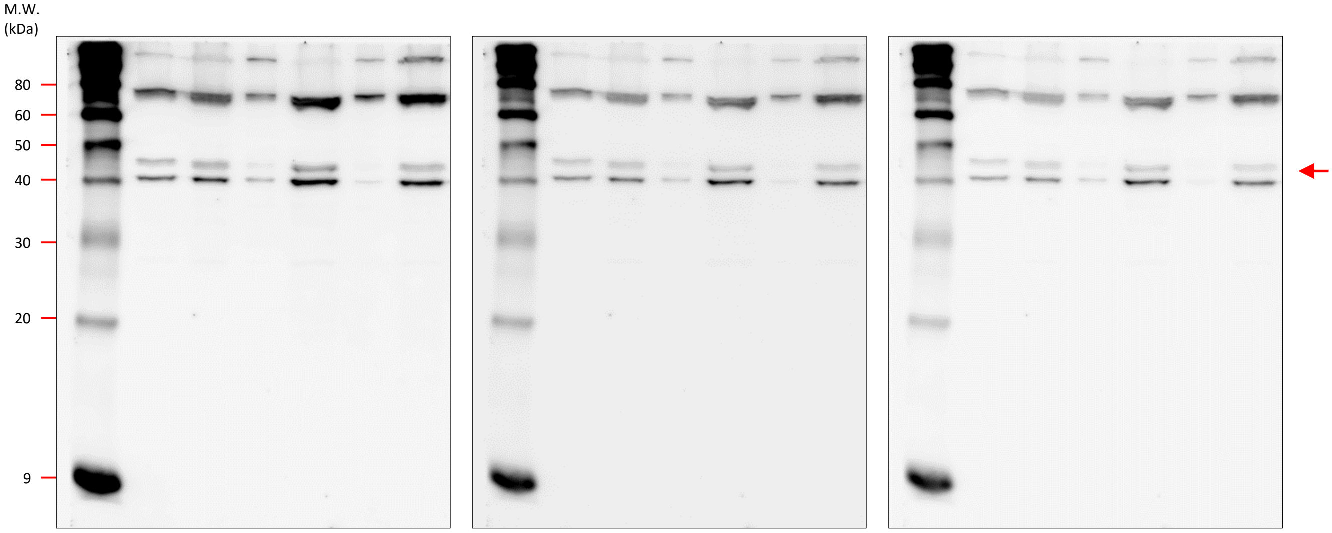

Figure 3E – NB4 XENOGRAFT MOUSE MODEL – p44/42 MAPK(Erk1/2) (42, 44 kDa)

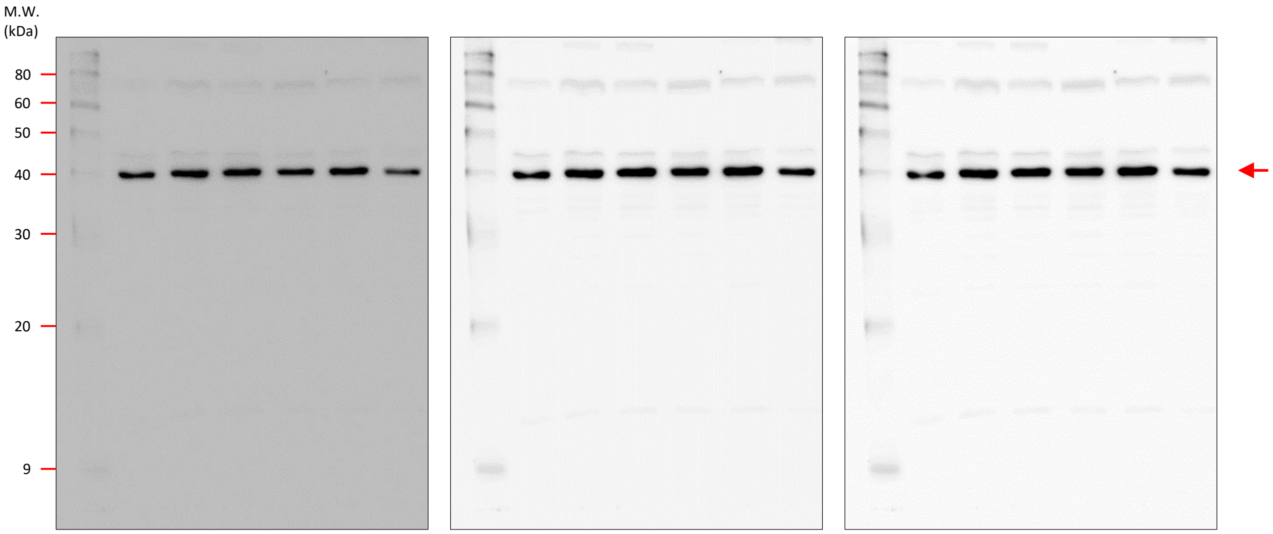

Figure 3E – NB4 XENOGRAFT MOUSE MODEL – Caspase-3 (35 kDa) and Cleaved Caspase-3 (19, 17 kDa)

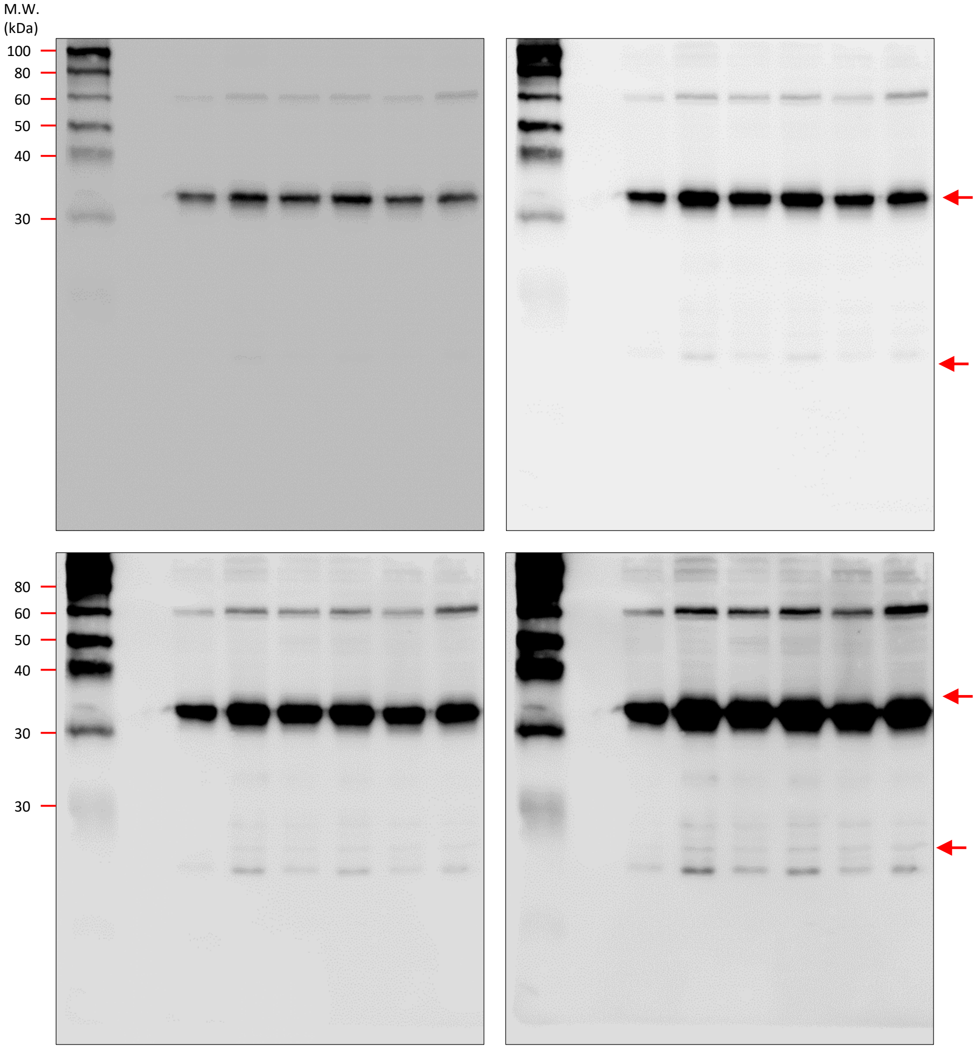

Figure 3E – NB4 XENOGRAFT MOUSE MODEL – β-actin (45 kDa)

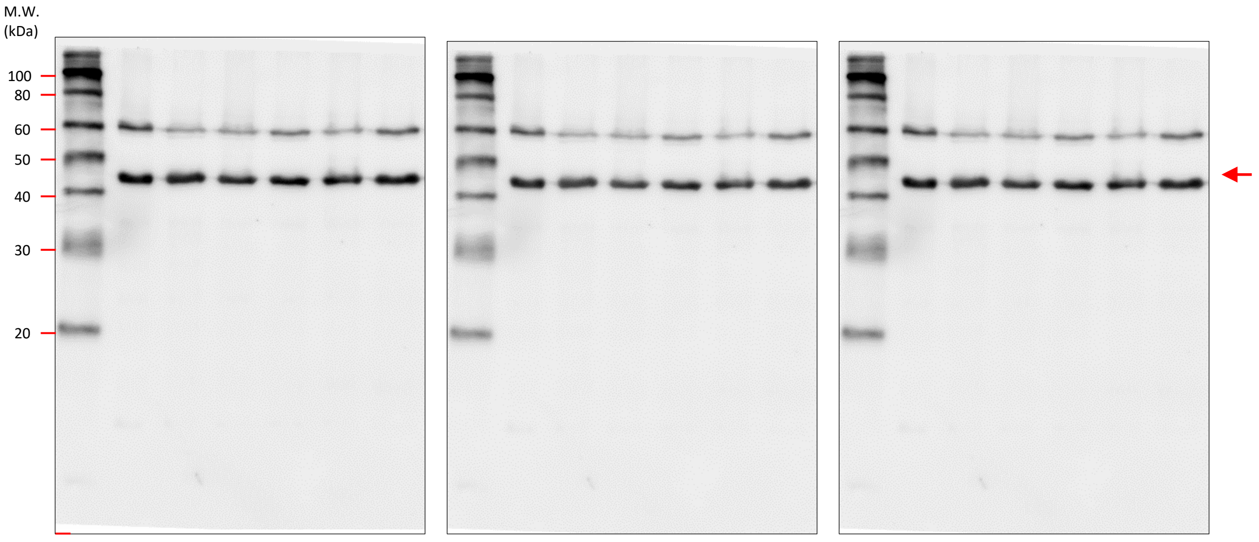

Supplement: Supplementary file 1 — Supplementary Data. [file 41598_2020_66223_MOESM1_ESM.pdf]
